# An Integrated Risk Function for Estimating the Global Burden of Disease Attributable to Ambient Fine Particulate Matter Exposure

**DOI:** 10.1289/ehp.1307049

**Published:** 2014-02-11

**Authors:** Richard T. Burnett, C. Arden Pope, Majid Ezzati, Casey Olives, Stephen S. Lim, Sumi Mehta, Hwashin H. Shin, Gitanjali Singh, Bryan Hubbell, Michael Brauer, H. Ross Anderson, Kirk R. Smith, John R. Balmes, Nigel G. Bruce, Haidong Kan, Francine Laden, Annette Prüss-Ustün, Michelle C. Turner, Susan M. Gapstur, W. Ryan Diver, Aaron Cohen

**Affiliations:** 1 Health Canada, Ottawa, Ontario, Canada; 2 Brigham Young University, Provo, Utah, USA; 3 MRC-PHE Centre for Environment and Health, School of Public Health, Imperial College London, London, UK; 4 School of Public Health, University of Washington, Seattle, Washington, USA; 5 Institute for Health Metrics and Evaluation, Seattle, Washington, USA; 6 Global Alliance for Clean Cookstoves, Washington, DC, USA; 7 Harvard School of Public Health, Harvard University, Cambridge, Massachusetts, USA; 8 U.S. Environmental Protection Agency, Research Triangle Park, North Carolina, USA; 9 School of Population and Public Health, University of British Colombia, Vancouver, British Columbia, Canada; 10 MRC-PHE Centre for Environment and Health, King’s College London, London, UK; 11 University of California, Berkeley, Berkeley, California, USA; 12 School of Medicine, University of California, San Francisco, San Francisco, California, USA; 13 School of Medicine, University of California, Berkeley, Berkeley, California, USA; 14 Department of Public Health and Policy, University of Liverpool, Liverpool, UK; 15 School of Public Health, Fudan University, Shanghai, China; 16 Exposure, Epidemiology, and Risk Program, Department of Environmental Health, Harvard School of Public Health, Boston, Massachusetts, USA; 17 World Health Organization, Geneva, Switzerland; 18 Institute of Population Health, University of Ottawa, Ottawa, Ontario, Canada; 19 American Cancer Society, Atlanta, Georgia, USA; 20 Health Effects Institute, Boston, Massachusetts, USA; * Senior Author

## Abstract

Background: Estimating the burden of disease attributable to long-term exposure to fine particulate matter (PM_2.5_) in ambient air requires knowledge of both the shape and magnitude of the relative risk (RR) function. However, adequate direct evidence to identify the shape of the mortality RR functions at the high ambient concentrations observed in many places in the world is lacking.

Objective: We developed RR functions over the entire global exposure range for causes of mortality in adults: ischemic heart disease (IHD), cerebrovascular disease (stroke), chronic obstructive pulmonary disease (COPD), and lung cancer (LC). We also developed RR functions for the incidence of acute lower respiratory infection (ALRI) that can be used to estimate mortality and lost-years of healthy life in children < 5 years of age.

Methods: We fit an integrated exposure–response (IER) model by integrating available RR information from studies of ambient air pollution (AAP), second hand tobacco smoke, household solid cooking fuel, and active smoking (AS). AS exposures were converted to estimated annual PM_2.5_ exposure equivalents using inhaled doses of particle mass. We derived population attributable fractions (PAFs) for every country based on estimated worldwide ambient PM_2.5_ concentrations.

Results: The IER model was a superior predictor of RR compared with seven other forms previously used in burden assessments. The percent PAF attributable to AAP exposure varied among countries from 2 to 41 for IHD, 1 to 43 for stroke, < 1 to 21 for COPD, < 1 to 25 for LC, and < 1 to 38 for ALRI.

Conclusions: We developed a fine particulate mass–based RR model that covered the global range of exposure by integrating RR information from different combustion types that generate emissions of particulate matter. The model can be updated as new RR information becomes available.

Citation: Burnett RT, Pope CA III, Ezzati M, Olives C, Lim SS, Mehta S, Shin HH, Singh G, Hubbell B, Brauer M, Anderson HR, Smith KR, Balmes JR, Bruce NG, Kan H, Laden F, Prüss-Ustün A, Turner MC, Gapstur SM, Diver WR, Cohen A. 2014. An integrated risk function for estimating the global burden of disease attributable to ambient fine particulate matter exposure. Environ Health Perspect 122:397–403; http://dx.doi.org/10.1289/ehp.1307049

## Introduction

Long-term exposure to ambient fine particulate matter (≤ 2.5 μg/m^3^ in aerodynamic diameter; PM_2.5_) is associated with increased mortality from nonaccidental and cause-specific diseases (Brook et al. 2010; Committee on the Medical Effects of Air Pollutants 2009; Cooke et al. 2007; Krewski et al. 2009). Epidemiologic cohort studies, conducted largely in the United States, have reported this association for annual ambient average concentrations from approximately 5 to 30 μg/m^3^, although definitive knowledge of which specific sources or characteristics of PM_2.5_ are responsible for these associations is currently lacking [U.S. Environmental Protection Agency (EPA) 2009; World Health Organization (WHO) 2006, 2007]. No epidemiologic study, however, has estimated the association of long-term exposure to direct measurements of PM_2.5_ with mortality from chronic cardiovascular and respiratory disease at the higher ambient exposures common in cities and other areas in Asia and other developing countries where annual average exposures can exceed 100 μg/m^3^ (Brauer et al. 2012; Health Effects Institute 2010). As a result, estimates of disease burden attributable to ambient air pollution in these locations have had to be based on extrapolations of the results of epidemiologic studies from locations with lower ambient PM_2.5_ exposures (Anenberg et al. 2010; Cohen et al. 2004; Evans et al. 2013).

Previous efforts to estimate global burden from exposure to ambient air pollution (AAP) in the form of PM_2.5_ postulated risk functions for cardiopulmonary mortality as linearly increasing in relative risk (RR) from 7.5 to 50 μg/m^3^, with no further change in RR at higher concentrations (Cohen et al. 2004). Sensitivity analyses included a model in which RR varied as the logarithm of concentration, producing a more gradual diminution of the marginal increase in RR than the base case model. The logarithmic model was subsequently recommended by the WHO for use in air pollution burden of disease estimates at the national level (Ostro 2004). The coefficients of these models were based on information from a single U.S. cohort study—the American Cancer Society Cancer Prevention Study II (CPS-II) (Krewski et al. 2009; Pope et al. 2002)—with exposure assignments of < 22 μg/m^3^. The form of the models used for global burden assessment was motivated largely by the concern that linear extrapolation using these coefficients would produce unrealistically large estimates of RR compared with other known PM_2.5_-related mortality risks such as active smoking (AS) and exposure to secondhand tobacco smoke (SHS) (Cohen et al. 2004; Ostro 2004). These RR models were also employed in more recent estimates of global mortality associated with ambient PM_2.5_ concentrations (Anenberg et al. 2010; Evans et al. 2013).

Absent empirical epidemiologic evidence on the magnitude of the association with mortality at high exposures of PM_2.5_ in ambient environments, Pope et al. (2011b) suggested that the integration of epidemiologic evidence on cardiovascular and lung cancer (LC) mortality RR from disparate types of PM_2.5_ exposure such as AAP, SHS, and AS, may provide insight into the shape of the exposure–response relation over a much wider range of exposures.

Here we present the methodology used to estimate the population attributable fraction (PAF) from exposure to ambient PM_2.5_ in the Global Burden of Diseases, Injuries, and Risk Factors Study 2010 (the GBD 2010 project) (Lim et al. 2012). We selected a mathematical form of the RR function with a PM_2.5_ concentration that could describe the observed relationships between RR and exposure for the five outcomes examined. We fit this model for cause-specific adult mortality for four causes of death—ischemic heart disease (IHD), stroke, chronic obstructive pulmonary disease (COPD), and LC—using RR information from epidemiologic studies of long-term exposure to particulate matter not only from AAP, SHS, and AS, but also from studies of household air pollution from solid cookfuel (household air pollution; HAP). We used these models to estimate the percentage of PAF associated with exposure to ambient PM_2.5_ for each of the 187 countries included in the GBD 2010 project. We identified a specific model form that best predicts source-specific RR for all four causes of death. In addition, we examined the relationship between PM_2.5_ exposure and the incidence of acute lower respiratory infection (ALRI) in infants, another health outcome considered in the GBD 2010 project. Because infants and young children are non(active)-smokers, the largest PM_2.5_ exposures considered for ALRI are from HAP.

## Methods

*Underlying assumptions*. The model we propose in here was based on the following underlying assumptions:

Exposure to PM_2.5_ from diverse combustion sources is associated with increased mortality from IHD, stroke, COPD, and LC and with increased incidence of ALRI. This assumption is based on systematic review of the available epidemiologic literature conducted by the GBD 2010 Ambient Air Pollution Expert Group as part of the GBD 2010 project (Lim et al. 2012).
The observed RRs from AAP, SHS, HAP, and AS are a function of PM_2.5_ mass inhaled concentration across all combustion particle sources (Smith 1987). The toxicity of PM_2.5_ is assumed to differ only with regard to inhaled mass (exposure) and not with PM_2.5_ composition. The toxicity of emissions from different combustion sources may well differ, but current knowledge does not allow definitive and quantifiable conclusions regarding their relative toxicity and little is known about international variation in source contributions around the world (Stanek et al. 2011; U.S. EPA 2009; WHO 2006).
The relation between PM_2.5_ exposure and excess mortality RR is not necessarily restricted to a linear function over the range of human exposure to PM_2.5_ from diverse sources (Pope et al. 2009, 2011b).
The RR of mortality from chronic disease experienced by people exposed to AAP, SHS, HAP, and AS is a function of long-term, cumulative exposure quantified in terms of daily average exposure concentration and does not depend on the temporal pattern of exposure (Pope et al. 2011a, 2011b). This assumption is required because the temporal nature of PM_2.5_ exposure differs for AAP, SHS, HAP, and AS.
The RR associated with each type of exposure does not depend on the other types of exposure. That is, we are assuming no interaction among the different exposure types for any cause of mortality. We are aware of no empirical epidemiologic evidence that tests that assumption; however, the direct epidemiologic evidence from the cohort studies we used to estimate the burden attributable to ambient PM_2.5_ shows that active cigarette smokers are also affected adversely by exposure to ambient PM_2.5_, and these studies do not provide support for significant heterogeneity of the relative excess AAP RR across smoking categories.

*Model form*. We selected a mathematical form of an integrated exposure–response (IER) model that could describe several patterns in RR thought to be *a priori* applicable to exposure–response models. We wanted the IER to be able to take shapes similar to models previously used for burden assessment, such as linear and log-linear (Cohen et al. 2004) and a power function (Pope et al. 2009, 2011b). In addition to these shapes, we also required the IER to have a property that it flattens out at high exposures, consistent with evidence of the relationship between IHD mortality and smoking intensity (Pope et al. 2009).

The form must equal 1 when PM_2.5_ values are below some concentration that represents a counterfactual low exposure where below this level there is no excess risk. We also desired a model that increases monotonically with increasing PM_2.5_ exposure concentration and could take a variety of shapes, such as near linear, sublinear, and supralinear. Our IER model has the following form:

for *z* < *z_cf_*, *RR*_IER_(*z*) = 1

for *z* ≥ *z_cf_*, *RR*_IER_(*z*) = 1 + α {1 – exp[– γ (*z – z_cf_*)^δ^]}, [1]

where *z* is the exposure to PM_2.5_ in micrograms per meter cubed and *z_cf_* is the counterfactual concentration below which we assumed there is no additional risk. For very large *z*, *RR*_IER_ approximates 1 + α. We included a power of PM_2.5_, δ, to predict risk over a very large range of concentrations. Further, *RR*_IER_ (*z_cf_* + 1) approximates 1 + αγ. Thus, γ = [*RR*_IER_ (*z_cf_* + 1) – 1]/[*RR*_IER_ (∞) – 1] can be interpreted as the ratio of the RR at low-to-high exposures. We term our model an “integrated-exposure response” model because its development requires the integration of exposures to PM_2.5_ from different combustion types (i.e., AAP, SHS, HAP, and AS).

In formulating our RR model, we relied on information on the RR of mortality at specified PM_2.5_ exposure concentrations from the available literature. Suppose we have a set of RR estimates {^^^*r_1_*^(^*^s^*^)^,…,^^^*r_Ks_*^(^*^s^*^)^, *s =* 1*,…,S*} and corresponding confidence intervals (CIs) based on PM_2.5_ concentrations {*z_1_*^(^*^s^*^)^,…,*z_Ks_*^(^*^s^*^)^, *s* = 1,…,*S*}, for S different types of PM_2.5_ sources, where *K_s_* is the number of RR estimates available from for source type S. The unknown parameters (α, γ, δ) are estimated by nonlinear regression methods. We then weighted the RR estimates by the inverse of the variance estimate of the logarithm of the RR in order to reflect the uncertainties in each estimate.

We compared the IER model to seven other models that have been previously suggested for burden assessment. These include an RR model that is linear in exposure throughout the global concentration range (Lin), a model that is linear up to 30 μg/m^3^ and constant above 30 μg/m^3^ (Lin30), a model that is linear up to 50 μg/m^3^ and constant above 50 μg/m^3^ (Lin50), and a model that is a function of the logarithm of exposure (Log). These models were used in a previous assessment of global burden of disease due to AAP exposure (Cohen et al. 2004). We also postulated a model in which we added an unknown parameter to concentration in the Log model to allow more flexibility in fitting the type-specific RRs (Log2). The sixth model examined related RR to a power of exposure as proposed by Pope et al. (2009, 2011b), with the seventh model equivalent to the IER with δ = 1(Exp). For the mathematical forms of the models, see the Supplemental Material (Sensitivity of RRs and PAFs to Model Form, pp. 9–11). We then calculated both the Akaike and Bayesian information criteria (AIC, BIC) for each of the eight models examined and five health outcomes as measures of goodness of fit.

The method of constructing uncertainty bounds on model predictions is described in detail in the Supplemental Material (Characterizing Uncertainty, pp. 28–29). Briefly, we simulated 1,000 sets of source type–specific RRs based on their point estimates and CIs and fit the IER model to these simulated values, obtaining 1,000 sets of parameter estimates of (α,γ,δ). Using these parameter estimates, we then generated 1,000 IER functions over the global concentration range. Estimates of uncertainty were also generated for the PM_2.5_ concentrations. Uncertainty in the PAFs is a function of the uncertainty in the IER model predictions and the exposure estimates and is determined by simulation methods as described in the Supplemental Material (Characterizing Uncertainty, pp. 28–29).

Specifics of the selection of source type–specific RR and PM_2.5_ exposure for each type are described below for the four mortality outcomes. The logarithm of the RR per micrograms per meter cubed, its SE, and associated PM_2.5_ concentration for the five outcomes is given by type of PM_2.5_ in Supplemental Material, Table S1.

*AAP*. To fit the risk models, we used cause-specific mortality AAP RR estimates from available published cohort studies. We evaluated each RR estimate at its study-specific PM_2.5_ mean concentration minus a less-polluted counterfactual level (Lim et al. 2012). Most RRs were obtained from published reports; however, in some cases new analyses were conducted for the present study. These estimates are identified in Supplemental Material, Table S1. We had eight studies reporting RR estimates for IHD mortality, five for stroke mortality, three for COPD mortality, and four for LC mortality.

*SHS*. We selected RRs for both IHD (8 studies reporting separate estimates for males and females) and LC (46 studies) mortality from studies included in the U.S. Surgeon General’s Report, *The Health Consequences of Involuntary Exposure to Tobacco Smoke* (Office on Smoking and Health 2006). We associated the RR of death due to SHS exposure with an equivalent ambient PM_2.5_ concentration of 20 μg/m^3^ for low-to-moderate SHS exposure and 50 μg/m^3^ for moderate-to-high exposure based on the analysis of Pope et al. (2009) for IHD mortality because RRs were reported by the Office on Smoking and Health (2006) for these two descriptive exposure categories. We assigned a concentration of 35 μg/m^3^ based on the midpoint of the range 20–50 μg/m^3^ for LC mortality because no specific description of the level of SHS exposure was provided by the Office on Smoking and Health (2006). We selected 29 RRs from studies examined by Oono et al. (2011) for stroke mortality on the basis of prospective cohort studies with an associated PM_2.5_ concentration of 35 μg/m^3^. There was insufficient evidence to estimate a RR due to SHS exposure for COPD mortality. We assumed that the SHS RRs are associated with a change in PM_2.5_ exposure based on nonsmoking subjects living with a smoker compared with those not living with a smoker. We have not incorporated other potential sources of PM_2.5_ exposure for these study subjects, such as from indoor sources, near-roadway conditions, or occupational exposures by subject.

*AS*. Following Pope et al. (2009, 2011b), we estimated the RR of each of the four causes of death for current cigarettes smoked per day compared with never smokers from the CPS-II. We estimated the RR and 95% CIs associated with 10 cigarettes-per-day groupings: 1–3, 4–7, 8–12, 13–17, 18–22, 23–27, 28–32, 33–37, 38–42, and > 42 cigarettes/day. We estimated that smoking a single cigarette was equivalent to breathing a daily ambient concentration of PM_2.5_ of 667 μg/m^3^, assuming an average breathing rate of 18 m^3^/day and an inhaled dose of 12,000 μg PM_2.5_ mass per cigarette (Pope et al. 2009). We then estimated the equivalent ambient concentration of PM_2.5_ by multiplying the average cigarettes/day smoked in each interval by 667 μg/m^3^. The shape of the curve fitted by Pope et al. (2009, 2011b) was not sensitive to the estimate of equivalent ambient PM_2.5_ concentrations for AS.

*HAP*. Smith et al. (2014) conducted a meta-analysis of studies examining COPD and LC incidence rates among men and women exposed to air pollution from burning coal or biomass for cooking. There were no studies relating IHD or stroke mortality or incidence to HAP at the time of the GBD 2010 project analyses, and thus this PM_2.5_ type cannot contribute to the fit of our RR function. The equivalent long-term PM_2.5_ exposure from HAP was estimated for study subjects using coal or biomass for cooking (Balakrishnan et al. 2013) and for those study subjects using cleaner fuels to integrate this information into our IER risk model. PM_2.5_ exposure estimates for women (300 μg/m^3^) were higher than for men (200 μg/m^3^). For the COPD meta-analysis, the relevant female control group was assumed to be using a mixture of gas and chimney stoves (an estimated PM_2.5_ exposure of 100 μg/m^3^). The PM_2.5_ exposure for males was estimated to be 65% of that for females (65 μg/m^3^). For LC, the female control group was assumed to be using only gas stoves with an estimated PM_2.5_ exposure of 70 μg/m^3^. For males, the exposure was again assumed to be 65% of females, resulting in an equivalent exposure of 45.5 μg/m^3^. The meta-analytic summary risk estimate for male COPD incidence in association with HAP PM_2.5_ was 1.90 (95% CI: 1.56, 2.32) and for females was 2.70 (95% CI: 1.95, 3.75). For LC incidence among males, the summary risk estimate was 1.26 (95% CI: 1.04, 1.52) and among females was 1.81 (95% CI: 1.07, 3.06).

The lower exposure estimates in the HAP studies are substantially higher than counterfactual exposure due to the nearby use of less clean fuels; therefore, these RRs are not directly comparable to those obtained from AAP, SHS, or AS types compared with either the counterfactual (i.e., AAP) or a 0-μg/m^3^ exposure (i.e., SHS, AS). This information was included in the curve-fitting process by equating the observed RRs to the ratio of the IER model evaluated at the respective two PM_2.5_ concentrations.

The HAP studies estimated effects on incidence rather than mortality. For building the IER, we assumed that the RRs of mortality and incidence are equal.

*Age-modification risk models for IHD and stroke mortality*. Epidemiologic studies of risk factors for both IHD and stroke indicate that the RR declines with the logarithm of age, reaching 1 between 100 and 120 years of age (Singh et al. 2013). We thus modified the type-specific RR for both IHD and stroke mortality using a linear regression model of the logarithm of the median age at death for each study with the intercept equal to 1 at 110 years of age. The slope of the regression line was estimated from a meta-analysis of several risk factors (Singh et al. 2013). We applied this age-modification to the RRs and fit the IER model for each age group separately.

*Selecting the counterfactual exposure*. For each risk factor examined in the GBD 2010 project (Lim et al. 2012), the distribution of exposure was compared with an alternative (counterfactual) distribution termed the theoretical-minimum-risk exposure distribution (TMRED). For AAP, zero exposure is not a practical counterfactual level because it is impossible to achieve even in pristine environments (Brauer et al. 2012). Furthermore, the lowest level of exposure to PM_2.5_ that is deemed beneficial has not been clearly identified. Defining the TMRED was based on two criteria (Lim et al. 2012): *a*) the availability of convincing evidence from epidemiologic studies that support a continuous reduction in risk of disease to the chosen distribution, and *b*) a distribution that is theoretically possible at the population level.

Lim et al. (2012) suggested that a positive counterfactual concentration be used. Their counterfactual concentration is bounded by the minimum concentrations observed in the studies used to estimate risk and some low percentile of the PM_2.5_ distribution. There is clearly no evidence of an association below observed levels, and it is impractical to estimate the shape of the curve at the extremes of the exposure distribution. Lim et al. (2012) suggested that the fifth percentile be used and that the lower and upper bounds on the counterfactual concentration be determined by the corresponding minimum and fifth percentiles, respectively, of the AAP PM_2.5_ exposure distribution for the CPS II cohort (Krewski et al. 2009), the largest cohort study of air pollution. The minimum was 5.8 μg/m^3^ and the fifth percentile was 8.8 μg/m^3^. Uncertainty in the counterfactual concentration was modeled as a uniform distribution between the minimum and fifth percentile.

*Estimation of PAF*. We estimated the PAF associated with ambient PM_2.5_ exposure for all 187 countries separately for 2005. We first estimated surface PM_2.5_ concentrations on a 0.1° × 0.1° grid for the globe using a combination of remote sensing and atmospheric models calibrated to ground monitoring data (Brauer et al. 2012). For each grid cell within a given country, we estimated the RR based on the IER model at the estimated PM_2.5_ concentration. We then constructed a population-weighted average RR for each country using the corresponding population count 0.1° × 0.1° grid cell (Brauer et al. 2012). Both the gridded PM_2.5_ and population values can be obtained from Brauer et al. (2012). The country-specific PAF = 1 – 1/*WRR*_IER_, where *WRR*_IER_ is the population-weighted average of the *RR*_IER_ values at each PM_2.5_ grid cell within the country.

*IER model for ALRI*. Mehta et al. (2013) reviewed the evidence for an association between exposure to ambient PM_2.5_ and ALRI. Four cohort studies were deemed appropriate to include in an IER model (Mehta et al. 2013). We included 23 studies of parental SHS and ALRI reported by the Office on Smoking and Health (2006) with each study-specific odds ratio (OR) assigned a PM_2.5_-equivalent ambient exposure of 50 μg/m^3^, assuming a moderate-to-high level of exposure. Smith et al. (2011) examined the relationship between exposure to carbon monoxide (CO) from the burning of solid biomass for heating and cooking and the incidence of ALRI in Guatemala and reported incidence rates by decile average of CO personal exposures. These decile CO averages were converted to PM_2.5_ concentrations using the following equation:

PM_2.5_(mg/m^–3^) = 0.10(0.093, 0.12) × CO(mg/m^–3^) + 0.067 (0.0069, 0.13), [2]

with 95% CIs displayed in parenthesis (Northcross et al. 2010). This equation had good predictive power (*R*^2^ = 0.76).

Incidence rates, *I*(*z_i_*), corresponding to the 10 decile values of PM_2.5_, denoted by *z_i_* for 1 = 1,…10, can be compared with the risk model by taking the ratio of incidence rates for all unique pairs of PM_2.5_ deciles, a total of 45 pairs, and equating them to the ratio of the corresponding risk model evaluated at the appropriate decile average. That is,

*RR*_ALRI_(*z_i_, z_j_*) = *I*(*z_i_*)/*I*(*z_j_*) = [1 + α{1 – exp[–γ (*z_i_ – z_cf_*)^δ^]}]  ÷ [1 + α{1 – exp[–γ (*z_j_ – z_cf_*)^δ^]}] [3]

for all 45 unique pairs of concentrations (*z_i_, z_j_*), ∀*i* > *j* = 1,…10. The 45 incidence rate ratios were combined with the 4 AAP cohort study ORs and the 23 SHS ORs in order to fit the IER model for ALRI. We assumed the same counterfactual uncertainty distribution as with the mortality IER models.

## Results

**Figure 1:**
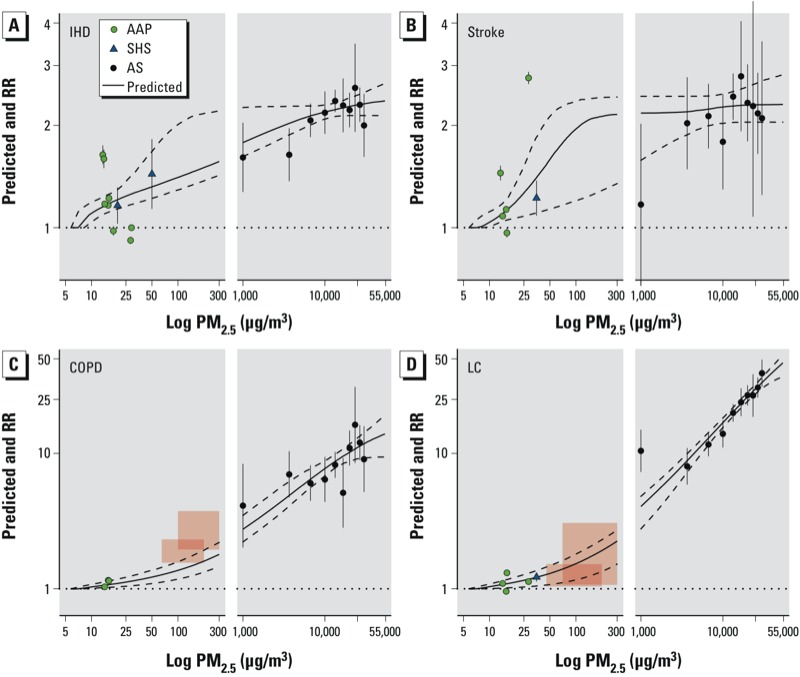
Predicted values of IER model (solid line) and 95% CIs (dashed line) and type-specific RRs (points) and 95% CIs (error bars) for IHD (*A*), stroke (*B*), COPD (*C*), and LC (*D*) mortality. Shaded boxes for COPD and LC mortality represent uncertainty (height) and exposure contrast (width) of RR HAP estimates for males (smaller boxes) and females (larger boxes) separately.

**Figure 2:**
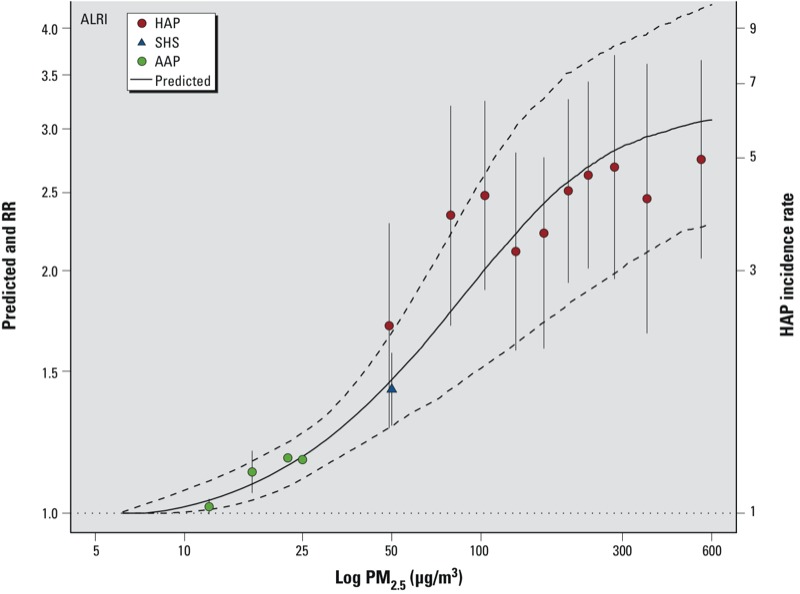
Predicted values of IER model (solid line) and 95% CIs (dashed line) and type-specific RRs (points) and 95% CIs (error bars) for ALRI in infants.

The average of the *RR*_IER_ predictions among the simulations are displayed for the four causes of death in Figure 1 in addition to the 95% CI and the type-specific RR estimates and corresponding 95% CIs used to fit the curves. The HAP RRs for COPD and LC are presented for males and females in Figure 1 as pink-shaded boxes with the height of each box representing the uncertainty in the RR estimates and the width representing the exposure contrast at which the RRs was assumed to pertain. Each box is centered at the RR estimate and the midpoint of the two exposure values. This alternate depiction of the HAP information was necessary because the lowest exposure levels were substantially higher than the counterfactual exposure and, therefore, not directly comparable to the RRs from the other sources. The pooled estimate of RR and its corresponding CI for SHS is displayed in placed of the study-specific SHS RRs for each unique PM_2.5_ value because the study-specific RRs and CI could not be visually distinguished. Results are presented similarly for ALRI in Figure 2. In addition to the RR, the incidence of ALRI is also displayed on the right-hand *y*-axis.

The *RR*_IER_ function fits well the RRs for all types of PM_2.5_ and causes of mortality, except for COPD and HAP, in which the IER model underestimates the observed RRs (Figure 1). This may be due to the use of the ratio of incidence rates rather than RR based on mortality data for this outcome. However, the IER curve fits the LC incidence data reasonably well. The time between diagnosis of COPD and mortality is much longer than that for LC, and thus the LC incidence data may better reflect mortality patterns than the COPD incidence data.

We compared the country-specific estimated PAFs using the age-modified models to those models using age-independent data. Age-modified *RR*_IER_ curves are displayed for IHD and stroke mortality in Supplemental Material, Figure S15 (top panels), with generally decreasing risk with increasing age. The country-specific PAFs based on risk models not modified by age and those in which age-modification models were used for both IHD and stroke mortality are presented in Supplemental Material, Figure S15 (bottom panels). Incorporating age-modification risk models tends to slightly decrease the PAF estimates.

**Figure 3:**
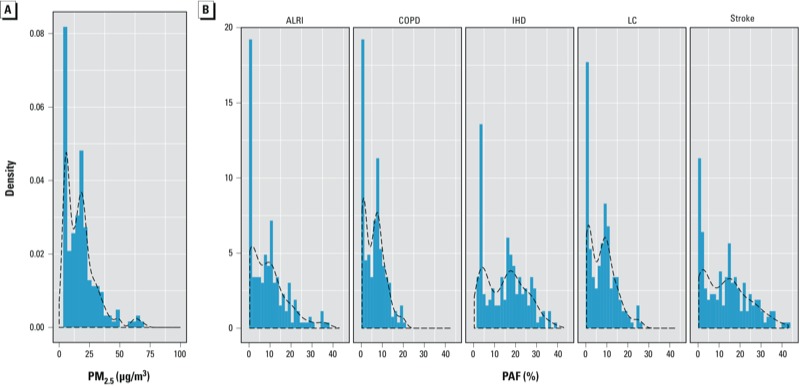
Density plots of country-specific, population-weighted PM_2.5_ concentrations (μg/m^3^) (*A*) and PAFs (*B*) by risk model and health outcome. Dashed lines represent smooth fit of density function.

The distribution of population-weighted country-average PM_2.5_ concentrations and PAFs are displayed in Figure 3. The country average PM_2.5_ concentrations ranged from 2–70 μg/m^3^ for 2005 (Figure 3A), whereas the country-level PAFs were < 0.4 for ALRI, IHD, and stroke and < 0.25 for LC and 0.2 for COPD (Figure 3B).

Plots similar to Figures 1 and 2 are displayed for the other seven model forms examined in Supplemental Material, Figures S1–S14 for both the four causes of death (Figures S1–S7) and ALRI (Figures S8–S14). In addition, both the AICs and BICs are given in Supplemental Material, Table S2, for all eight models and five outcomes. The IER model was a better predictor of the type-specific RRs than the other seven models examined for ALRI and three of the four causes of death. For COPD mortality, the Power model provided a better fit than the IER model on the basis of lower AIC and BIC values (see Supplemental Material, Table S2). This was likely due to the better prediction of the HAP RR, for which the IER model clearly underestimated the RR. Graphical comparisons of the predicted values to the type-specific RRs in Supplemental Material, Figures S1–S14, verify the conclusions drawn from the AIC/BIC results.

## Discussion

Exposure to PM_2.5_ in ambient air has been linked to an increased risk of death from chronic cardiovascular and respiratory disease and LC in cohort studies in the United States and Europe (Chen et al. 2008; U.S. EPA 2009). Unfortunately, few long-term cohort studies have been reported for these diseases in other regions such as East and South Asia and the Middle East, where ambient exposures are much higher and where the relative contribution of specific sources of air pollution differ from those in North America and Europe (Brauer et al. 2012; Heath Effects Institute 2010).

To derive the shape of the exposure–response curve at higher ambient concentrations, we incorporated information on risk due to exposure to SHS, HAP, and AS in order to extend the risk estimates to higher exposures. The IER model combines information on mortality RR from separate types of combustion, unified by equating the delivered dose from all types in terms of equivalent ambient PM_2.5_ exposures. Although we assumed that the toxicity of PM_2.5_ exposure, as characterized by RR, changes with the magnitude of exposure, we also assumed that at any fixed exposure level, toxicity is roughly equivalent among all types and temporal patterns of PM_2.5_ exposure. These are important assumptions because estimated PM_2.5_ exposure throughout the world, whether from ambient origin or household indoor combustion, has not been differentiated by the components or sources of fine particulate matter.

Only evidence from multiple epidemiologic studies of long-term exposure to PM_2.5_ in highly polluted settings can provide definitive estimates of the shape of the exposure–response function for mortality from chronic cardiovascular and respiratory diseases. However, these are starting to appear. For example, Cao et al. (2011) reported an increased risk of mortality from cardiovascular and respiratory disease and LC associated with long-term exposure to total suspended particulates (TSPs) in 71,000 residents of 31 Chinese cities. Their study offers an opportunity to assess the ability of our *RR*_IER_ model to estimate the observed RRs in situations with very high levels of outdoor air pollution. In order to estimate PM_2.5_ RRs in the cohort, the authors used a 3:1 ratio to convert TSP to PM_2.5_, based on current and historical Chinese data (Cao et al. 2011). Estimated PM_2.5_ (converted from TSP) concentrations ranged among cities from 38 to 166 μg/m^3^. Increases of 2.1% (95% CI: –0.3%, 4.6%), 3.3% (95% CI: 0.9%, 5.4%), and 3.3% (95% CI: –0.3%, 6.9%) in IHD, stroke, and LC mortality, respectively, were associated with a 10-μg/m^3^ change in estimated equivalent PM_2.5_ exposures in this cohort (Kan H, personal communication).

Because the cohort members did not experience exposures near the lowest concentrations applicable to our RR model (i.e., the counterfactual concentration), we cannot determine RRs estimated from the cohort and directly compare them to our RR model, which is relative to a much lower counterfactual concentration. However, we can determine RR between concentrations observed in the cohort itself. We first determined the mean of the four quartiles of PM_2.5_ concentrations as 40, 91, 106, and 127 μg/m^3^, respectively (Kan H, personal communication) and calculated the RR between consecutive quartile averages assuming the exponential risk model form as was used by the study authors. The geometric average of these three RRs was then determined as a summary measure of change in risk over the PM_2.5_ exposure distribution. A similar calculation was undertaken for the *RR*_IER_ model. The RRs observed in the Chinese cohort and those predicted by *RR*_IER_ were similar for the three causes of death examined [IHD: China RR = 1.06 (95% CI: 0.99, 1.14) and IER RR = 1.05 (95% CI: 1.03, 1.1); stroke: China RR = 1.10 (95% CI: 1.03, 1.17) and IER RR = 1.08 (95% CI: 1.01, 1.14; LC: China RR = 1.10 (95% CI: 0.99, 1.22) and IER RR = 1.09 (95% CI: 1.06, 1.12)], suggesting that our IER model yielded reasonable predictions in the change in risk over a range of concentrations that prevail in China and other highly polluted settings that were not observed in cohort studies conducted in North America and Western Europe.

There are, however, some limitations in this comparison. First, TSP was a poorer predictor of cardiovascular mortality than PM_2.5_ in U.S.-based cohort studies (Pope et al. 2002). Second, uncertainty about the temporal and spatial consistency of the TSP/PM_2.5_ conversion ratio of 3:1 added uncertainty to our interpretation of the results from the Chinese cohort.

Additional uncertainties are due to a lack of information on actual exposure to PM_2.5_ for some source-specific RRs used to fit the model, notably *a*) scarce information on actual exposure from SHS in the relevant epidemiologic studies (Pope et al. 2009, 2011b), which required the estimation of PM_2.5_ concentrations from other studies; *b*) potential misclassification of exposure for SHS estimates due to possible co-exposure from AAP of the exposed group; and *c*) the duration of exposure, which differs when it comes to exposures from AAP, SHS, HAP, and AS—the lifetime duration of exposure in AS may be much shorter than in the other exposures and the received doses may, therefore, not be proportional to concentrations according to type of exposure. Uncertainties may be reduced by improving precision in the actual exposure estimates of the RRs from the epidemiologic literature used for developing the proposed model.

Multiple studies were used to estimate RRs associated with exposure to AAP, SHS, and HAP. For AS, we estimated RRs for active cigarette smokers from a single cohort, the CPS II. This cohort was also used in the GBD 2010 project to estimate risk specifically for AS (Lim et al. 2012). However, the pattern of the association between the number of cigarettes smoked per day and cause-specific mortality observed in the CPS-II cohort may not reflect the patterns observed in other cohort studies of AS (e.g., Pirie et al. 2013). Similarly, the IER for ALRI is fit through RR from studies of AAP and SHS conducted in a limited number of mostly high-income countries, and a single developing country RR estimate for HAP PM_2.5_ exposure and ALRI (Smith et al. 2011). We thus recommend that future work on the IER function include additional sensitivity analyses of the type-specific RRs to which the curve is fit. Future work could also include the uncertainty in the estimate of PM_2.5_ from CO and new information in this relationship (McCracken et al. 2013).

The key assumptions that underlie the IER, discussed above, largely serve to justify the integration of risk estimates for different types of PM exposure. These assumptions, and their tenability, have been addressed elsewhere (Pope et al. 2009, 2011a, 2011b). Unfortunately, for several of the most critical assumptions, those concerning the relative toxicity per unit mass of PM_2.5_ of different types (e.g., AAP and AS), not accounting for the temporal pattern of exposure, and the absence of interaction among types of combustion, there is little empirical evidence against which to evaluate those assumptions or to evaluate in detail specific implications of their violation. Each warrants additional research.

Although we set the counterfactual concentration to be drawn from a uniform distribution with a lower bound of 5.8 μg/m^3^ and an upper bound of 8.8 μg/m^3^, we are not suggesting that there is convincing evidence that PM_2.5_ mortality and ALRI risk is zero below any specific concentration based on biological considerations (Brook et al. 2010). Absence of such evidence from epidemiologic studies does not necessarily imply evidence of the absence of such a counterfactual concentration. We thus take the conservative approach and set a positive counterfactual concentration. However, our approach can be adapted to a different counterfactual if new evidence supporting a positive association at lower concentrations becomes available. One such piece of evidence was observed in Canada, where positive associations as low as 2 μg/m^3^ were noted (Crouse et al. 2012).

The Lin50 and Log models proposed by Cohen et al. (2004) were used for the previous GBD estimates, and the Log model is currently recommended by the WHO (Ostro 2004). However, the unknown parameters in these models were estimated from a single cohort study of AAP, the CPS-II, which required analysis of the original data. The IER model uses RR estimates available in the open literature, allowing periodic updating of risk functions based on systematic review of the literature, and it does not require analyses of primary data not in the public domain. As new epidemiologic studies and evidence on type-specific PM_2.5_ exposure appear, the models can be reestimated by any interested member of the scientific community using publically available information.

## Conclusion

Fine particulate mass–based RR models can be developed that cover the entire global range of ambient exposure to PM_2.5_ by integrating RR information from different combustion sources that generate emissions of particulate matter. The specific RR model form we identified in the present study can provide superior predictive power for leading global causes of mortality for air pollution compared with a range of alternative model forms.

## Supplemental Material

(856 KB) PDF
