# Erratum: “An Integrated Risk Function for Estimating the Global Burden of Disease Attributable to Ambient Fine Particulate Matter Exposure”

**DOI:** 10.1289/ehp.122-A235

**Published:** 2014-09-01

In “An Integrated Risk Function for Estimating the Global Burden of Disease Attributable to Ambient Fine Particulate Matter Exposure” by Burnett et al. [Environ Health Perspect 122:397–403 (2014); http://dx.doi.org/10.1289/ehp.1307049], the authors omitted a reference. Balakrishnan et al. (2013) should have been cited in the first paragraph of “Methods” for HAP (household air pollutants). The correct sentence and reference are provided below.

The equivalent long-term PM_2.5_ exposure from HAP was estimated for study subjects using coal or biomass for cooking (Balakrishnan et al. 2013) and for those study subjects using cleaner fuels to integrate this information into our IER risk model.

Balakrishnan K, Ghosh S, Ganguli B, Sambandam S, Bruce NG, Barnes DF, et al. 2013. State and national household concentrations of PM_2.5_ from solid cookfuel use: results from measurements and modeling in India for estimation of the global burden of disease. Environ Health 12:77; doi:10.1186/1476-069X-12-77.

The authors regret the error.

