# Supplementary material for: An Integrated Risk Function for Estimating the Global Burden of Disease Attributable to Ambient Fine Particulate Matter Exposure
Source: Environ Health Perspect. 2014 Feb 11;122(4):397–403. doi: 10.1289/ehp.1307049 (PMC3984213; doi:10.1289/ehp.1307049)
Supplement: (856 KB) PDF [file ehp.1307049.s001.pdf]

Environmental Health Perspectives doi:10.1289/ehp.1307049

Supplemental Material

An Integrated Risk Function for Estimating the Global Burden of Disease Attributable to Ambient Fine Particulate Matter Exposure

Richard T. Burnett, C. Arden Pope III, Majid Ezzati, Casey Olives, Stephen S. Lim, Sumi Mehta, Hwashin H. Shin, Gitanjali Singh, Bryan Hubbell, Michael Brauer, H. Ross Anderson, Kirk R. Smith, John R. Balmes, Nigel G. Bruce, Haidong Kan, Francine Laden, Annette Prüss-Ustün, 3Michelle C. Turner, Susan M. Gapstur, W. Ryan Diver, and Aaron Cohen

Table of Contents **Page**

Table S1. 2

Sensitivity of RRs and PAFs to model form 9

Figure S1–S14 12

Table S2. 26

Characterizing Uncertainty 28

Uncertainty in the estimates of (α, β, δ) 28

Uncertainty in the exposure estimate of $PM_{2.5}$ 28

Uncertainty in the counterfactual concentration 29

Uncertainty in the population attributable risk 29

Age-modification risk models for ischemic heart disease and stroke mortality 29

Figure S15. 31

References 32

Table S1. Logarithm of relative risks and standard errors per µg/m^3 of $PM_{2.5}$ by outcome and type of combustion used to fit Integrated Exposure-Response Model. If RR obtained from published source reference is given. RR obtained from original analysis indicated under Study Reference. Denominator concentration used for COPD and LC HAPs RR and ALRI incidence rates.

**Study Reference**	**Outcome**	**$PM_{2.5}$ Combustion Type**	**Logarithm of RR/µg/m^3**	**Standard Error**	**$PM_{2.5}$ Concentration Used to Evaluate RR**	**$PM_{2.5}$ Denominator Concentration**
Original analysis based on ACS	IHD	AAP	0.023111	0.00443	14.2	
Lepeule et al. 2012	IHD	AAP	0.028518	0.007865	15.9	
Lipsett et al. 2011	IHD	AAP	0.018232	0.007701	15.6	
Puett et al. 2009	IHD	AAP	0.07031	0.032196	13.9	
Miller et al. 2007	IHD	AAP	0.079299	0.03236	13.5	
Chen et al. 2005	IHD	AAP	0	0.007118	29	
Puett et al. 2011	IHD	AAP	-0.00202	0.016446	17.9	
Beelen et al. 2008	IHD	AAP	-0.00408	0.012412	28.3	
Svendsen et al. 1987 (Males)	IHD	SHS	-0.10536	1.483197	20	
Hole et al. 1989 (Males)	IHD	SHS	0.737164	0.634965	20	
Hirayama et al. 1990 (Males)	IHD	SHS	0.076961	0.093807	20	
La Vecchia et al. 1993 (Males)	IHD	SHS	0.122218	0.468175	20	
He et al. 1994 (Males)	IHD	SHS	0.476234	0.60933	20	
Steenland et al. 1996 (Males)	IHD	SHS	0.270027	0.108203	20	
Ciruzzi et al. 1998 (Males)	IHD	SHS	0.215111	0.361876	20	
Rosenlund et al. 2001 (Males)	IHD	SHS	0.019803	0.169737	20	
Svendsen et al. 1987 (Females)	IHD	SHS	1.166271	0.720849	50	
Hole et al. 1989 (Females)	IHD	SHS	1.415853	0.625511	50	
Hirayama et al. 1990 (Females)	IHD	SHS	0.262364	0.105034	50	
La Vecchia et al. 1993 (Females)	IHD	SHS	0.262364	0.489011	50	
He et al. 1994 (Females)	IHD	SHS	1.269761	0.754263	50	
Steenland et al. 1996 (Females)	IHD	SHS	0.131028	0.082431	50	
Ciruzzi et al. 1998 (Females)	IHD	SHS	1.393766	0.714909	50	
Rosenlund et al. 2001 (Females)	IHD	SHS	0.457425	0.247568	50	
Pope et al. 2011	IHD	AS	0.476234	0.119648	1000	
Pope et al. 2011	IHD	AS	0.494696	0.091361	3667	

Study Reference	Outcome	$PM_{2.5}$ Combustion Type	Logarithm of RR/μg/m^3	Standard Error	$PM_{2.5}$ Concentration Used to Evaluate RR	$PM_{2.5}$ Denominator Concentration
Pope et al. 2011	IHD	AS	0.727549	0.058031	6667	
Pope et al. 2011	IHD	AS	0.779325	0.073388	10000	
Pope et al. 2011	IHD	AS	0.858662	0.038824	13333	
Pope et al. 2011	IHD	AS	0.828552	0.092984	16667	
Pope et al. 2011	IHD	AS	0.797507	0.059757	20000	
Pope et al. 2011	IHD	AS	0.947789	0.152309	23333	
Pope et al. 2011	IHD	AS	0.832909	0.059647	26667	
Pope et al. 2011	IHD	AS	0.693147	0.108631	30000	
Original analysis based on ACS	STROKE	AAP	0.011333	0.004982	14.2	
Lepeule et al. 2012	STROKE	AAP	-0.00408	0.015987	15.9	
Lipsett et al. 2011	STROKE	AAP	0.014842	0.011407	15.6	
Miller et al. 2007	STROKE	AAP	0.060432	0.025364	13.4	
Beelen et al. 2008	STROKE	AAP	0.048243	0.021029	28.3	
Gillis et al. 1984 (Males)	STROKE	SHS	-1.10866	1.087418	35	
Gillis et al. 1984 (Females)	STROKE	SHS	0.631272	1.093869	35	
Sandler et al. 1989 (Males)	STROKE	SHS	-0.03046	0.206434	35	
Sandler et al. 1989 (Females)	STROKE	SHS	0.215111	0.094188	35	
Yamada et al. 2003 (Males)	STROKE	SHS	0.122218	0.904277	35	
Yamada et al. 2003 (Females)	STROKE	SHS	-0.06188	0.255197	35	
Iribarren et al. 2004 (Males)	STROKE	SHS	0.019803	0.187381	35	
Iribarren er al. 2004 (Females)	STROKE	SHS	0.157004	0.124706	35	
Whincup et al. 2004 (Males)	STROKE	SHS	0.431782	0.41577	35	
Qureshi et al. 2005 (Females)	STROKE	SHS	-0.10536	0.197242	35	
Wen et al. 2006 (Females)	STROKE	SHS	0.41871	0.175639	35	
Hill et al. 2007 (Males)	STROKE	SHS	0.463734	0.168868	35	
Hill et al. 2007 (Females)	STROKE	SHS	-0.10536	0.15079	35	
Hill et al. 2007 (Males)	STROKE	SHS	0.598837	0.213399	35	
Hill et al. 2007 (Females)	STROKE	SHS	0.157004	0.222774	35	
Glymour et al. 2008 (Males)	STROKE	SHS	0.48858	0.300914	35	
Glymour et al. 2008 (Females)	STROKE	SHS	0.378436	0.198807	35	
Glymour et al. 2008 (Males)	STROKE	SHS	0.565314	0.15551	35	
Glymour et al. 2008 (Females)	STROKE	SHS	0.444686	0.314322	35	

Study Reference	Outcome	$PM_{2.5}$ Combustion Type	Logarithm of RR/μg/m^3	Standard Error	$PM_{2.5}$ Concentration Used to Evaluate RR	$PM_{2.5}$ Denominator Concentration
Jefferis et al. 2010	STROKE	SHS	-0.06188	0.083547	35	
Original analysis based on ACS	STROKE	AS	0.157004	0.279005	1000	
Original analysis based on ACS	STROKE	AS	0.708036	0.159101	3667	
Original analysis based on ACS	STROKE	AS	0.756122	0.111752	6667	
Original analysis based on ACS	STROKE	AS	0.582216	0.164769	10000	
Original analysis based on ACS	STROKE	AS	0.887891	0.080345	13333	
Original analysis based on ACS	STROKE	AS	1.026042	0.190406	16667	
Original analysis based on ACS	STROKE	AS	0.845868	0.132851	20000	
Original analysis based on ACS	STROKE	AS	0.824175	0.382641	23333	
Original analysis based on ACS	STROKE	AS	0.774727	0.140318	26667	
Original analysis based on ACS	STROKE	AS	0.741937	0.265557	30000	
Original analysis based on ACS	COPD	AAP	0.004879	0.005314	14.2	
Lepeule et al. 2012	COPD	AAP	0.0157	0.015748	15.9	
Lipsett et al. 2011	COPD	AAP	0.017395	0.015568	15.6	
Smith et al. 2014	COPD	HAP	0.641854	0.255426	200	65
Smith et al. 2014	COPD	HAP	0.993	0.167	330	100
Original analysis based on ACS	COPD	AS	1.413423	0.362642	1000	
Original analysis based on ACS	COPD	AS	1.94591	0.200793	3667	
Original analysis based on ACS	COPD	AS	1.796747	0.154106	6667	
Original analysis based on ACS	COPD	AS	1.859418	0.195083	10000	
Original analysis based on ACS	COPD	AS	2.107786	0.112795	13333	
Original analysis based on ACS	COPD	AS	1.631199	0.302756	16667	
Original analysis based on ACS	COPD	AS	2.393339	0.151127	20000	
Original analysis based on ACS	COPD	AS	2.788093	0.330657	23333	
Original analysis based on ACS	COPD	AS	2.48574	0.152194	26667	
Original analysis based on ACS	COPD	AS	2.203869	0.285136	30000	
Original analysis based on ACS	LC	AAP	0.013103	0.003795	14.2	
Lepeule et al. 2012	LC	AAP	0.031481	0.014427	15.9	
Lipsett et al. 2011	LC	AAP	-0.00513	0.015034	15.6	
Beelen et al. 2008	LC	AAP	0.005827	0.013279	28.3	
Brownson et al. 1992	LC	SHS	0.518794	0.745212	35	
Chan and Fung 1982	LC	SHS	-0.28768	0.282228	35	

Study Reference	Outcome	$PM_{2.5}$ Combustion Type	Logarithm of RR/µg/m³	Standard Error	$PM_{2.5}$ Concentration Used to Evaluate RR	$PM_{2.5}$ Denominator Concentration
Correa et al, 1983	LC	SHS	0.727549	0.476773	35	
Garfnkel et al. 1985	LC	SHS	0.239017	0.191404	35	
Geng et al. 1987	LC	SHS	0.770108	0.353055	35	
Inoue and Hirayama 1987	LC	SHS	0.936093	0.634192	35	
Kabat et al. 1995	LC	SHS	0.076961	0.299366	35	
Kabat et al. Wynder 1984	LC	SHS	-0.23572	0.58224	35	
Kalandidi et al. 1990	LC	SHS	0.641854	0.326769	35	
Ko et al. 1997	LC	SHS	0.262364	0.324736	35	
Lam et al. 1987	LC	SHS	0.698135	0.313149	35	
Lee et al. 2000	LC	SHS	0	0.507959	35	
Liu et al. 1993	LC	SHS	0.506818	0.419499	35	
Shimizu et al. 1988	LC	SHS	0.076961	0.266613	35	
Sobue 1990	LC	SHS	0.122218	0.188021	35	
Trichopoulos et al. 1983	LC	SHS	0.875469	0.396262	35	
Zaridze et al. 1998	LC	SHS	0.425268	0.187429	35	
Akiba et al. 1986	LC	SHS	0.405465	0.260625	35	
Boffetta et al. 1999	LC	SHS	0.10436	0.116617	35	
Brownson et al. 1987	LC	SHS	0	0.103435	35	
Bufler et a. 1984	LC	SHS	-0.22314	0.438945	35	
Fontham et al. 1994	LC	SHS	0.254642	0.109894	35	
Gao et al. 1987	LC	SHS	0.173953	0.190452	35	
Humble et al. 1987	LC	SHS	0.587787	0.662055	35	
Koo et al. 1997	LC	SHS	0.494696	0.323325	35	
Lam et al. 1987	LC	SHS	0.500775	0.180101	35	
Liu et al. 1991	LC	SHS	-0.26136	0.478805	35	
Nyberg et al. 1997	LC	SHS	0.04879	0.288623	35	
Pershagen et al. 1987	LC	SHS	0.182322	0.280258	35	
Shen et al. 1998	LC	SHS	0.48858	0.444916	35	
Stockwell et al. 1992	LC	SHS	0.470004	0.345547	35	
Svenson et al. 1989	LC	SHS	0.231112	0.406965	35	
Wang et al. 1996	LC	SHS	0.10436	0.270932	35	
Wu et al. 1985	LC	SHS	0.182322	0.36406	35	

**Study Reference**	**Outcome**	**$PM_{2.5}$ Combustion Type**	**Logarithm of RR/μg/m^3**	**Standard Error**	**$PM_{2.5}$ Concentration Used to Evaluate RR**	**$PM_{2.5}$ Denominator Concentration**
Wu et al. 1990	LC	SHS	-0.23572	0.124486	35	
Butler et al. 1998	LC	SHS	0.703098	0.867882	35	
Cardenas et al. 1997	LC	SHS	0.182322	0.20687	35	
Garfunkel et al. 1985	LC	SHS	0.182322	0.192289	35	
Hirayama 1981	LC	SHS	0.336472	0.176823	35	
Hole et al. 1989	LC	SHS	0.693147	1.128787	35	
Johnson et al. 2001	LC	SHS	0.48858	0.376507	35	
Wang et al. 2000	LC	SHS	0.173953	0.267812	35	
Seow et al. 2002	LC	SHS	0.262364	0.176823	35	
Smith et al. 2014	LC	HAP	0.231112	0.096809	200	45.5
Smith et al. 2014	LC	HAP	0.593	0.268	330	70
Pope et al. 2011	LC	AS	2.345645	0.182696	1000	
Pope et al. 2011	LC	AS	2.083185	0.158627	3667	
Pope et al. 2011	LC	AS	2.453588	0.102988	6667	
Pope et al. 2011	LC	AS	2.634045	0.118683	10000	
Pope et al. 2011	LC	AS	2.989714	0.075685	13333	
Pope et al. 2011	LC	AS	3.170526	0.120746	16667	
Pope et al. 2011	LC	AS	3.289148	0.088681	20000	
Pope et al. 2011	LC	AS	3.285412	0.185462	23333	
Pope et al. 2011	LC	AS	3.42198	0.087763	26667	
Pope et al. 2011	LC	AS	3.667656	0.117077	30000	
Brauer et al. (2002)	ALRI	AAP	0.012257	0.030595	16.9	
Hertz-Picciotto et al. (2007)	ALRI	AAP	0.010495	0.003981	22.3	
Karr et al. (2007)	ALRI	AAP	0.008618	0.002288	25	
Karr et al. (2009)	ALRI	AAP	0.003922	0.010991	12.1	
Blizzard et al. 2003	ALRI	SHS	0.463734	0.147312	50	
Bonu et al. 2004	ALRI	SHS	0.139762	0.075314	50	
Etiler et al. 2002	ALRI	SHS	0.067659	0.148168	50	
Kristensen et al. 2006	ALRI	SHS	0.371564	0.14942	50	
Baker et al. 2006	ALRI	SHS	0.254642	0.12521	50	
Broor et al. 2001	ALRI	SHS	-1.7148	1.064895	50	
Chen et al. 1994	ALRI	SHS	0.398776	0.174405	50	

Study Reference	Outcome	$PM_{2.5}$ Combustion Type	Logarithm of RR/μg/m^3	Standard Error	$PM_{2.5}$ Concentration Used to Evaluate RR	$PM_{2.5}$ Denominator Concentration
Duijts et al. 2008	ALRI	SHS	-0.19845	0.274887	50	
Ekwo et al. 1983	ALRI	SHS	0.737164	0.317623	50	
Ferris et al. 1985	ALRI	SHS	0.615186	0.087697	50	
Forastiere et al. 1992	ALRI	SHS	0.277632	0.112884	50	
Gardner et al. 1984	ALRI	SHS	0.223144	0.22149	50	
Kock et al. 2003	ALRI	SHS	0.756122	0.248502	50	
Margolis et al. 1997	ALRI	SHS	0.336472	0.207783	50	
Nuesslein et al. 1999	ALRI	SHS	0.076961	0.941041	50	
Ogston et al. 1985	ALRI	SHS	0.662688	0.368793	50	
Ogston et al. 1987	ALRI	SHS	0.518794	0.115821	50	
Pedreira et al. 1985	ALRI	SHS	0.239017	0.13706	50	
Rylander et al. 1995	ALRI	SHS	0.774727	0.257175	50	
Taylor et al. 1987	ALRI	SHS	0.378436	0.104149	50	
Hassan et al. 2001	ALRI	SHS	0.770108	0.213568	50	
Suzuki et al. 2009	ALRI	SHS	0.438255	0.109485	50	
Victora et al. 1994	ALRI	SHS	-0.06188	0.134529	50	
Smith et al. 2011	ALRI	HAP	0.268264	0.447947	79	49
Smith et al. 2011	ALRI	HAP	0.589684	0.325059	103	49
Smith et al. 2011	ALRI	HAP	0.334639	0.329612	131	49
Smith et al. 2011	ALRI	HAP	0.41961	0.364845	163	49
Smith et al. 2011	ALRI	HAP	0.610787	0.327824	197	49
Smith et al. 2011	ALRI	HAP	0.682406	0.312955	230	49
Smith et al. 2011	ALRI	HAP	0.718465	0.345909	282	49
Smith et al. 2011	ALRI	HAP	0.575364	0.397693	363	49
Smith et al. 2011	ALRI	HAP	0.753269	0.325687	553	49
Smith et al. 2011	ALRI	HAP	0.32142	0.436931	103	79
Smith et al. 2011	ALRI	HAP	0.066375	0.431997	131	79
Smith et al. 2011	ALRI	HAP	0.151346	0.455861	163	79
Smith et al. 2011	ALRI	HAP	0.342523	0.433673	197	79
Smith et al. 2011	ALRI	HAP	0.414142	0.426658	230	79
Smith et al. 2011	ALRI	HAP	0.450201	0.436423	282	79
Smith et al. 2011	ALRI	HAP	0.3071	0.485299	363	79

Study Reference	Outcome	$PM_{2.5}$ Combustion Type	Logarithm of RR/µg/m^3	Standard Error	$PM_{2.5}$ Concentration Used to Evaluate RR	$PM_{2.5}$ Denominator Concentration
Smith et al. 2011	ALRI	HAP	0.485005	0.444977	553	79
Smith et al. 2011	ALRI	HAP	-0.25505	0.333622	131	103
Smith et al. 2011	ALRI	HAP	-0.17007	0.343254	163	103
Smith et al. 2011	ALRI	HAP	0.021103	0.307069	197	103
Smith et al. 2011	ALRI	HAP	0.092722	0.312731	230	103
Smith et al. 2011	ALRI	HAP	0.128781	0.327205	282	103
Smith et al. 2011	ALRI	HAP	-0.01432	0.373858	363	103
Smith et al. 2011	ALRI	HAP	0.163585	0.317819	553	103
Smith et al. 2011	ALRI	HAP	0.084971	0.343366	163	131
Smith et al. 2011	ALRI	HAP	0.276148	0.306331	197	131
Smith et al. 2011	ALRI	HAP	0.347767	0.312737	230	131
Smith et al. 2011	ALRI	HAP	0.383826	0.350659	282	131
Smith et al. 2011	ALRI	HAP	0.240725	0.382238	363	131
Smith et al. 2011	ALRI	HAP	0.41863	0.323761	553	131
Smith et al. 2011	ALRI	HAP	0.191177	0.329942	197	163
Smith et al. 2011	ALRI	HAP	0.262796	0.337799	230	163
Smith et al. 2011	ALRI	HAP	0.298855	0.373284	282	163
Smith et al. 2011	ALRI	HAP	0.155755	0.399347	363	163
Smith et al. 2011	ALRI	HAP	0.333659	0.351879	553	163
Smith et al. 2011	ALRI	HAP	0.071619	0.289433	230	197
Smith et al. 2011	ALRI	HAP	0.107678	0.322166	282	197
Smith et al. 2011	ALRI	HAP	-0.03542	0.355146	363	197
Smith et al. 2011	ALRI	HAP	0.142482	0.312631	553	197
Smith et al. 2011	ALRI	HAP	0.036059	0.334963	282	230
Smith et al. 2011	ALRI	HAP	-0.10704	0.377171	363	230
Smith et al. 2011	ALRI	HAP	0.070863	0.3172	553	230
Smith et al. 2011	ALRI	HAP	-0.1431	0.398081	363	282
Smith et al. 2011	ALRI	HAP	0.034804	0.336203	553	282
Smith et al. 2011	ALRI	HAP	0.177905	0.386069	553	363

Sensitivity of RRs and PAFs to model form

We considered seven alternative RR models that have been suggested in the literature or variations on these previous examined forms. The first alternative risk model assumes a linear increase in RR up to 50 μg/m^3 and no change in RR above this level. This model was used by Cohen et al. (2004) to estimate the burden of disease from cardiopulmonary mortality due to outdoor urban air pollution exposures in 2000 (WHO 2002):

for $z < z_{cf}$, $RR_{Lin50}(z) = 1$,

for $z_{cf} < z \leq 50$, $RR_{Lin50}(z) = 1 + \gamma\,(z - z_{cf})$, and

for $z > 50$, $RR_{Lin50}(z) = 1 + \gamma\,(50 - z_{cf})$. [1]

The second model form assumes a linear increase in RR up to 30 μg/m^3 as this concentration is the highest value reported in any of the AAP cohort studies:

for $z < z_{cf}$, $RR_{Lin30}(z) = 1$,

for $z_{cf} < z \leq 30$, $RR_{Lin30}(z) = 1 + \gamma\,(z - z_{cf})$, and

for $z > 30$, $RR_{Lin30}(z) = 1 + \gamma\,(30 - z_{cf})$. [2]

The third model assumes that the RR increases as the logarithm of $PM_{2.5}$ exposure on the exponential scale (Cohen et al. 2004; Ostro 2004):

for $z < z_{cf}$, $RR_{Log}(z) = 1$,

for $z \geq z_{cf}$, $RR_{Log}(z) = [(z + 1) / (z_{cf} + 1)]^{\rho}$. [3]

This model can be made more flexible by adding an additional parameter, ϕ, which alters the rate of increase of the risk function at low concentrations:

for $z < z_{cf}$, $RR_{Log2}(z) = 1$,

for $z \geq z_{cf}$, $RR_{Log2}(z) = [(z + \phi) / (z_{cf} + \phi)]^{\rho}$. [4]

The fifth model assumes a power function form proposed by Pope et al. (2009, 2011):

for $z < z_{cf}$, $RR_{Power}(z) = 1$,

for $z \geq z_{cf}$, $RR_{Power}(z) = 1 + \theta(z - z_{cf})^{\eta}$. [5]

The sixth model considered is a linear model:

for $z < z_{cf}$, $RR_{Linear}(z) = 1$,

for $z \geq z_{cf}$, $RR_{Linear}(z) = 1 + \tau(z - z_{cf})$. [6]

The seventh and final alternative model is the RR_{IER} model with the power of concentration δ set to unity and thus has the form:

for $z < z_{cf}$, $RR_{Exp}(z) = 1$

for $z \geq z_{cf}$, $RR_{Exp}(z) = 1 + \alpha\{1 - \exp[-\gamma (z - z_{cf})]\}$. [7]

Predicted RRs and CIs from the seven alternative model forms examined are presented in Figures S1-S7 for the four causes of death and in Figures S8-S14 for ALRI. We calculated both the Akaike (AIC) and Bayesian (BIC) Information Criteria for each of the eight models

examined as a measure of goodness-of-fit of the various forms of risk models. These measures include different penalties for the number of estimated parameters. These values are presented in Table S2 with smaller (or larger negative) values indicating better fit. The RR_{IER} model returned the lowest AIC and BIC of the eight models considered for four of the five outcomes examined (Table S1). Only for COPD was the IER model not the best fit. The *Log2* model provided a slightly better fit by 0.4% for the AIC and 0.5% for the BIC compared the IER model (Table S1). The *Linear*, *Lin30,* and *Lin50* models clearly under/over predict the input RRs for various ranges of $PM_{2.5}$ concentrations (Figurers S1-S14). The *Exp and Log* models tended to be the next best predictors. The *Power* and *Log2* models gave similar and generally superior predictions to the other model forms except for the IER model which was superior to all other models for four of the five outcomes examined and nearly as good with the fifth outcome (COPD). We conclude from this analyses that the single model form best predicting all five outcomes was the IER model.

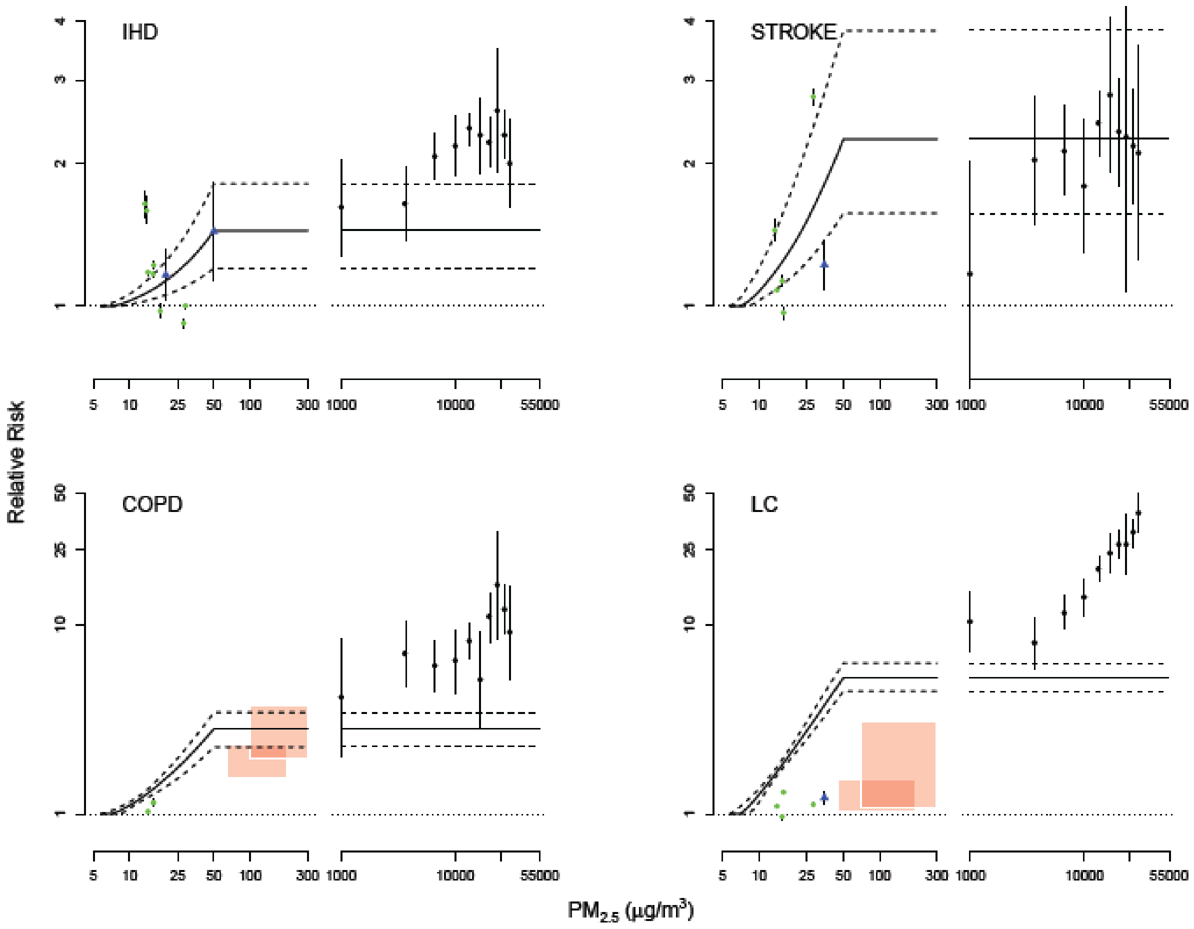

Figure S1. Predicted values of *Lin50* model (solid line) and 95% confidence interval (dashed line) for ischemic heart disease (IHD), stroke, chronic obstructive pulmonary disease (COPD), and lung cancer (LC) mortality. Type-specific relative risks (RR) (points) and 95% confidence intervals (error bars) also presented. Green dots represent AAP cohort study RRs, blue triangles represent pooled SHS RRs, and black dots represent AS RRs. Shaded boxes for COPD and LC mortality represent uncertainty (height) and exposure contrast (width) of RR HAP estimates for males and females separately. $PM_{2.5}$ concentrations are on the logarithmic (base 10) scale on x-axis.

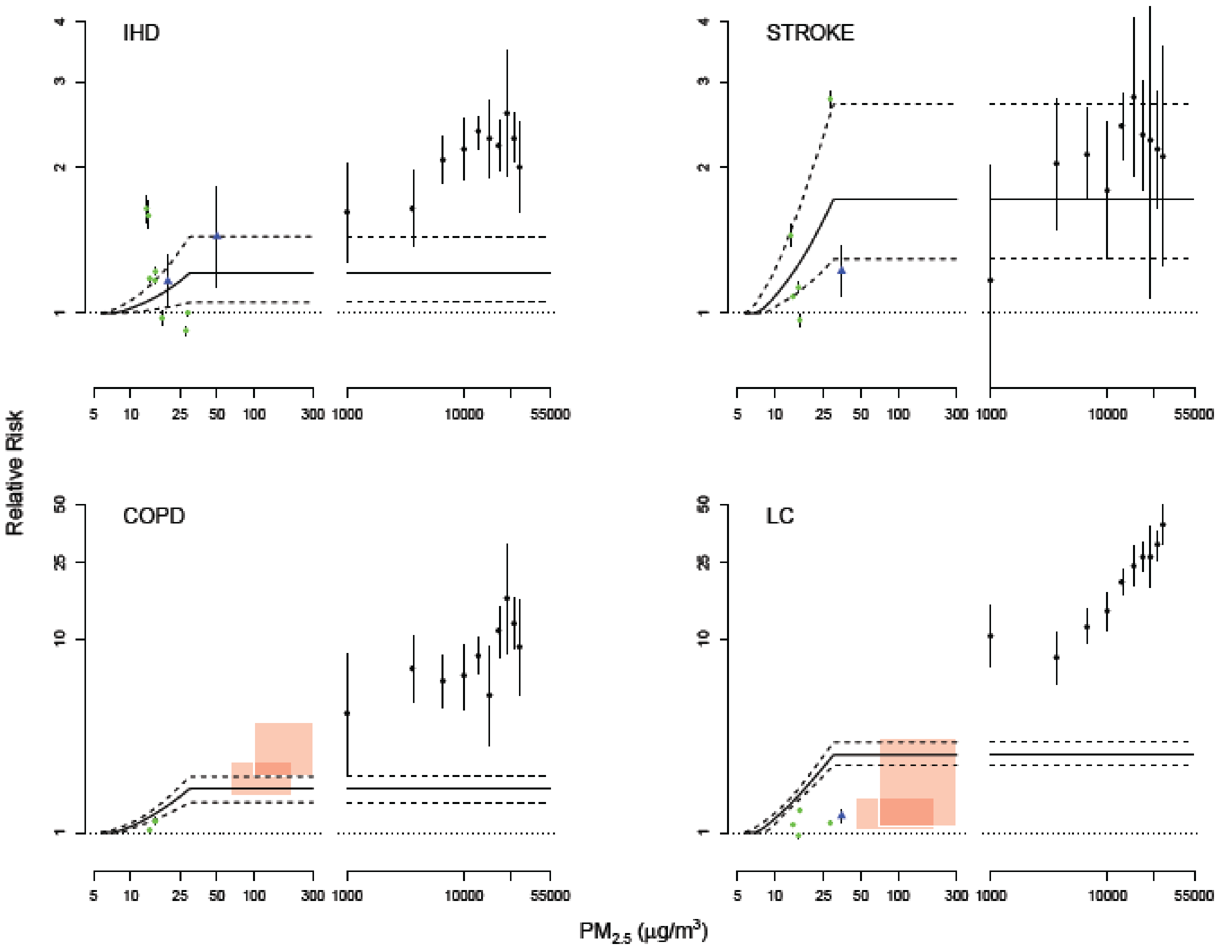

Figure S2. Predicted values of *Lin30* model (solid line) and 95% confidence interval (dashed line) for ischemic heart disease (IHD), stroke, chronic obstructive pulmonary disease (COPD), and lung cancer (LC) mortality. Type-specific relative risks (RR) (points) and 95% confidence intervals (error bars) also presented. Green dots represent AAP cohort study RRs, blue triangles represent pooled SHS RRs, and black dots represent AS RRs. Shaded boxes for COPD and LC mortality represent uncertainty (height) and exposure contrast (width) of RR HAP estimates for males and females separately. $PM_{2.5}$ concentrations are on the logarithmic (base 10) scale on x-axis.

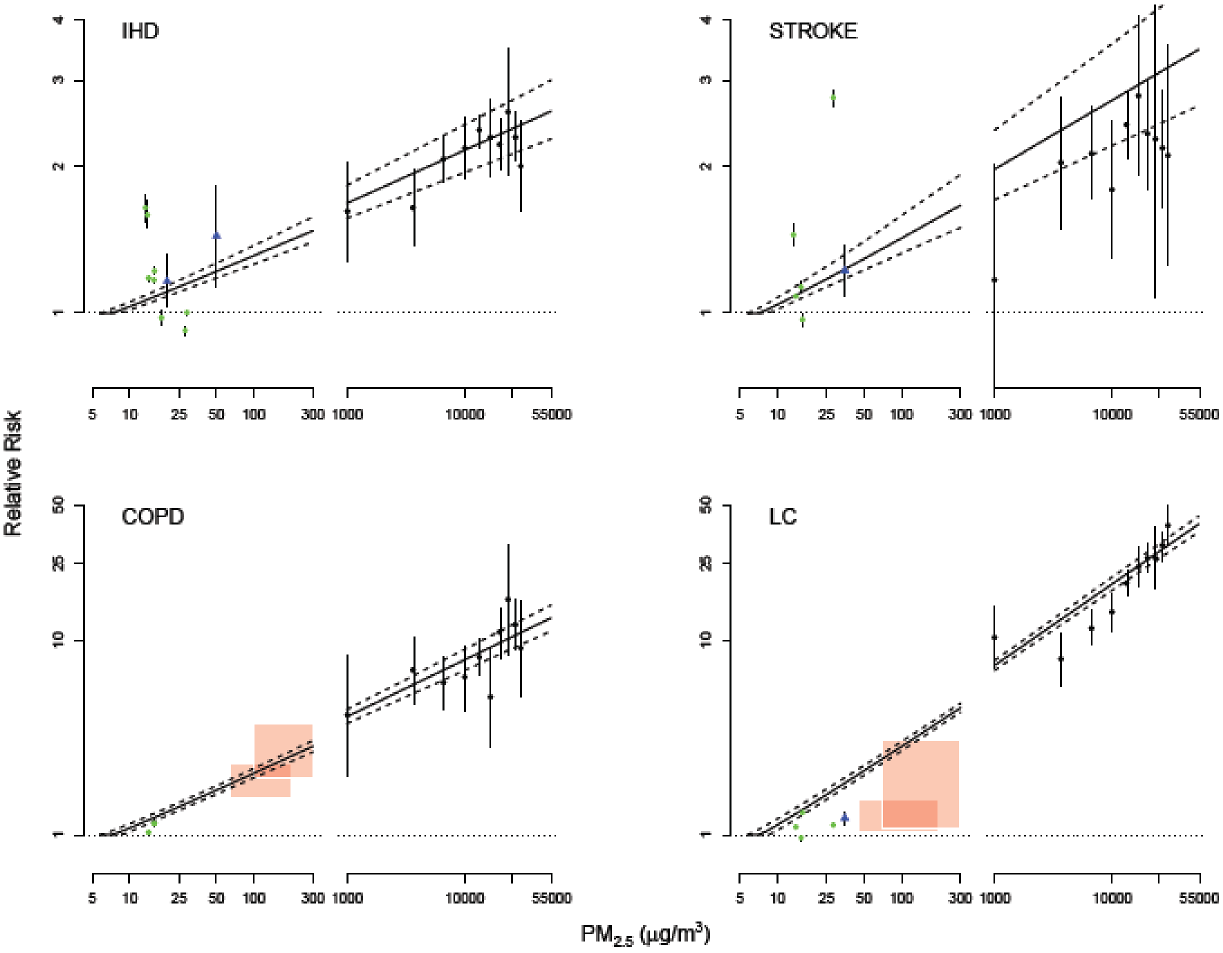

Figure S3. Predicted values of *Log* model (solid line) and 95% confidence interval (dashed line) for ischemic heart disease (IHD), stroke, chronic obstructive pulmonary disease (COPD), and lung cancer (LC) mortality. Type-specific relative risks (RR) (points) and 95% confidence intervals (error bars) also presented. Green dots represent AAP cohort study RRs, blue triangles represent pooled SHS RRs, and black dots represent AS RRs. Shaded boxes for COPD and LC mortality represent uncertainty (height) and exposure contrast (width) of RR HAP estimates for males and females separately. $PM_{2.5}$ concentrations are on the logarithmic (base 10) scale on x-axis.

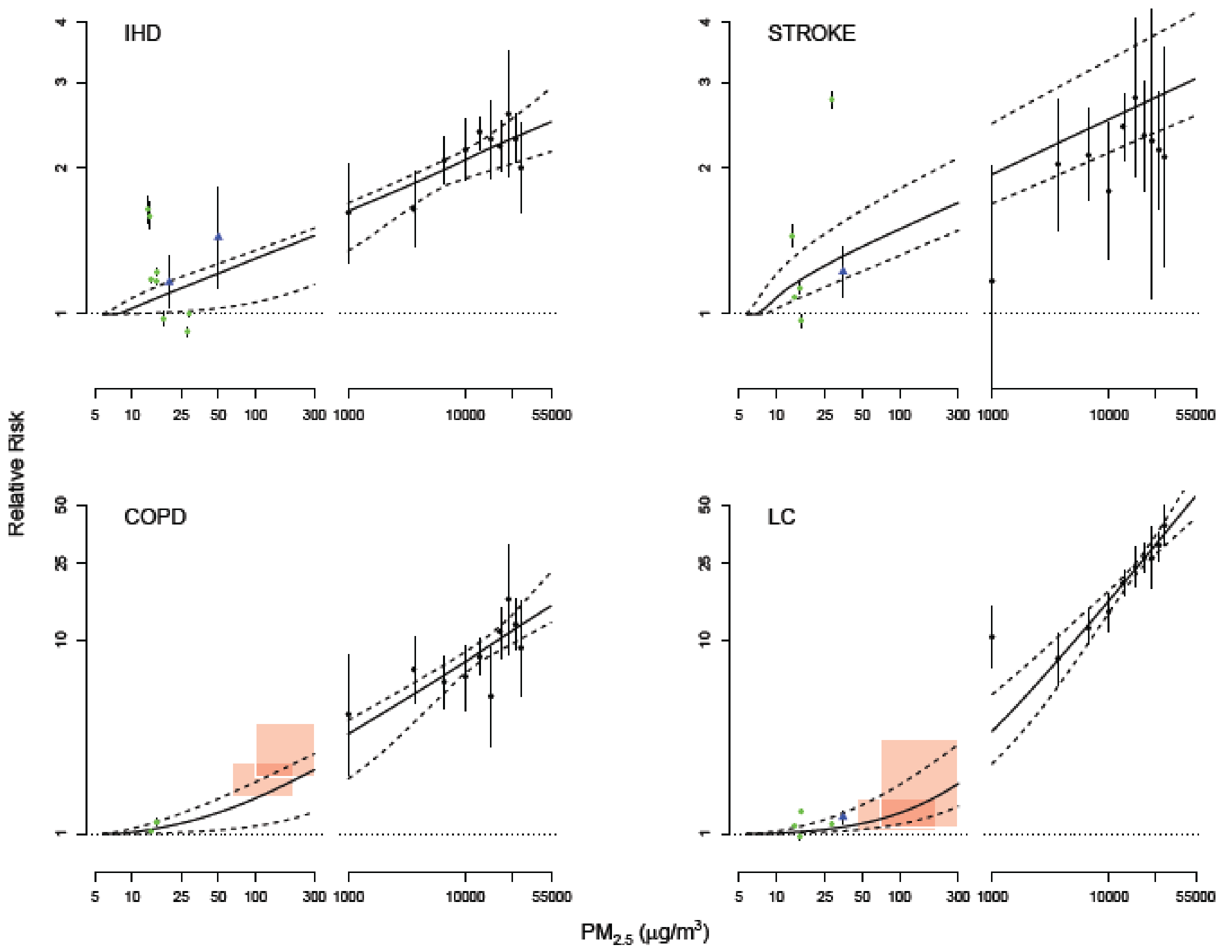

Figure S4. Predicted values of *Log2* model (solid line) and 95% confidence interval (dashed line) for ischemic heart disease (IHD), stroke, chronic obstructive pulmonary disease (COPD), and lung cancer (LC) mortality. Type-specific relative risks (RR) (points) and 95% confidence intervals (error bars) also presented. Green dots represent AAP cohort study RRs, blue triangles represent pooled SHS RRs, and black dots represent AS RRs. Shaded boxes for COPD and LC mortality represent uncertainty (height) and exposure contrast (width) of RR HAP estimates for males and females separately. $PM_{2.5}$ concentrations are on the logarithmic (base 10) scale on x-axis.

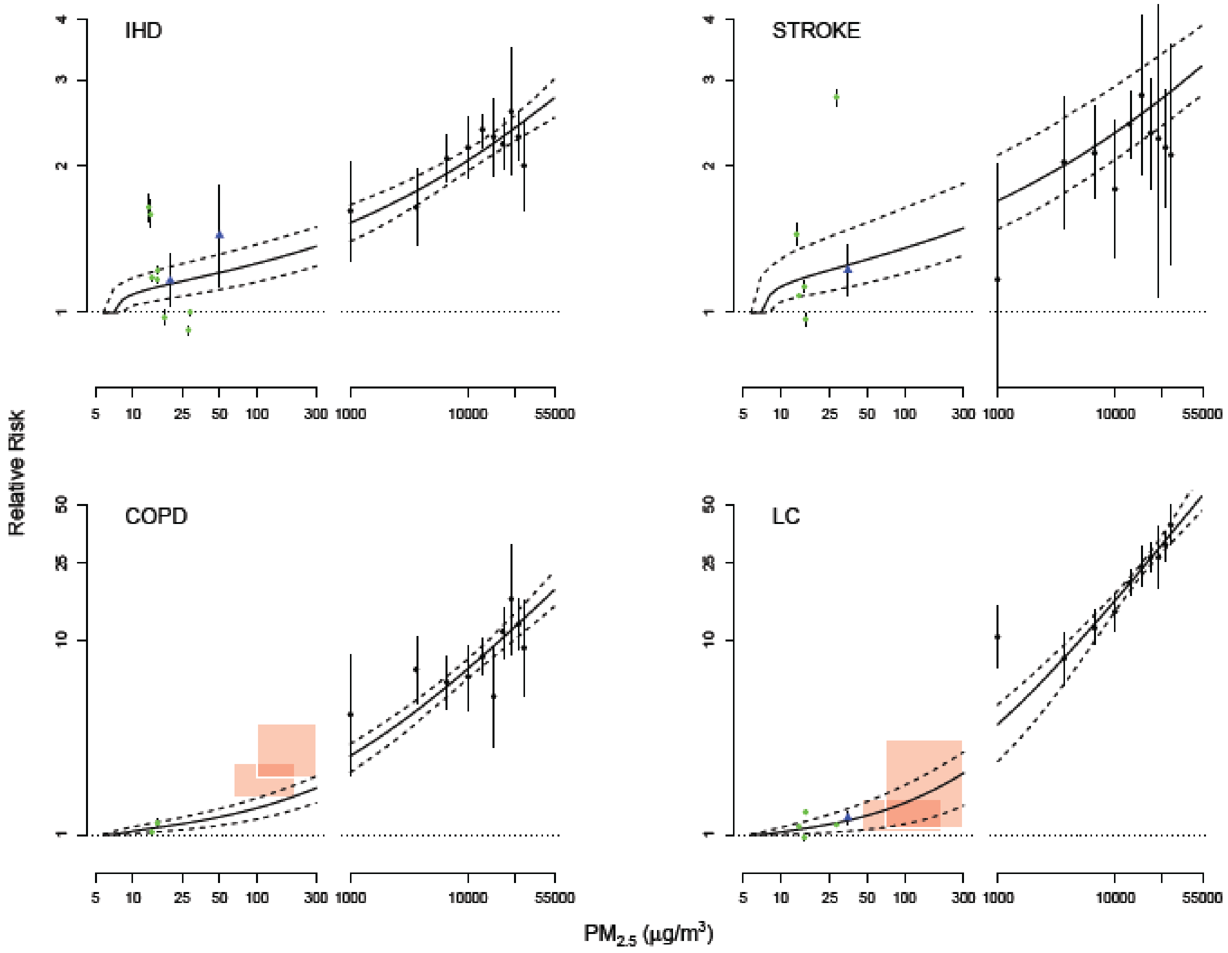

Figure S5. Predicted values of *Power* model (solid line) and 95% confidence interval (dashed line) for ischemic heart disease (IHD), stroke, chronic obstructive pulmonary disease (COPD), and lung cancer (LC) mortality. Type-specific relative risks (RR) (points) and 95% confidence intervals (error bars) also presented. Green dots represent AAP cohort study RRs, blue triangles represent pooled SHS RRs, and black dots represent AS RRs. Shaded boxes for COPD and LC mortality represent uncertainty (height) and exposure contrast (width) of RR HAP estimates for males and females separately. $PM_{2.5}$ concentrations are on the logarithmic (base 10) scale on x-axis.

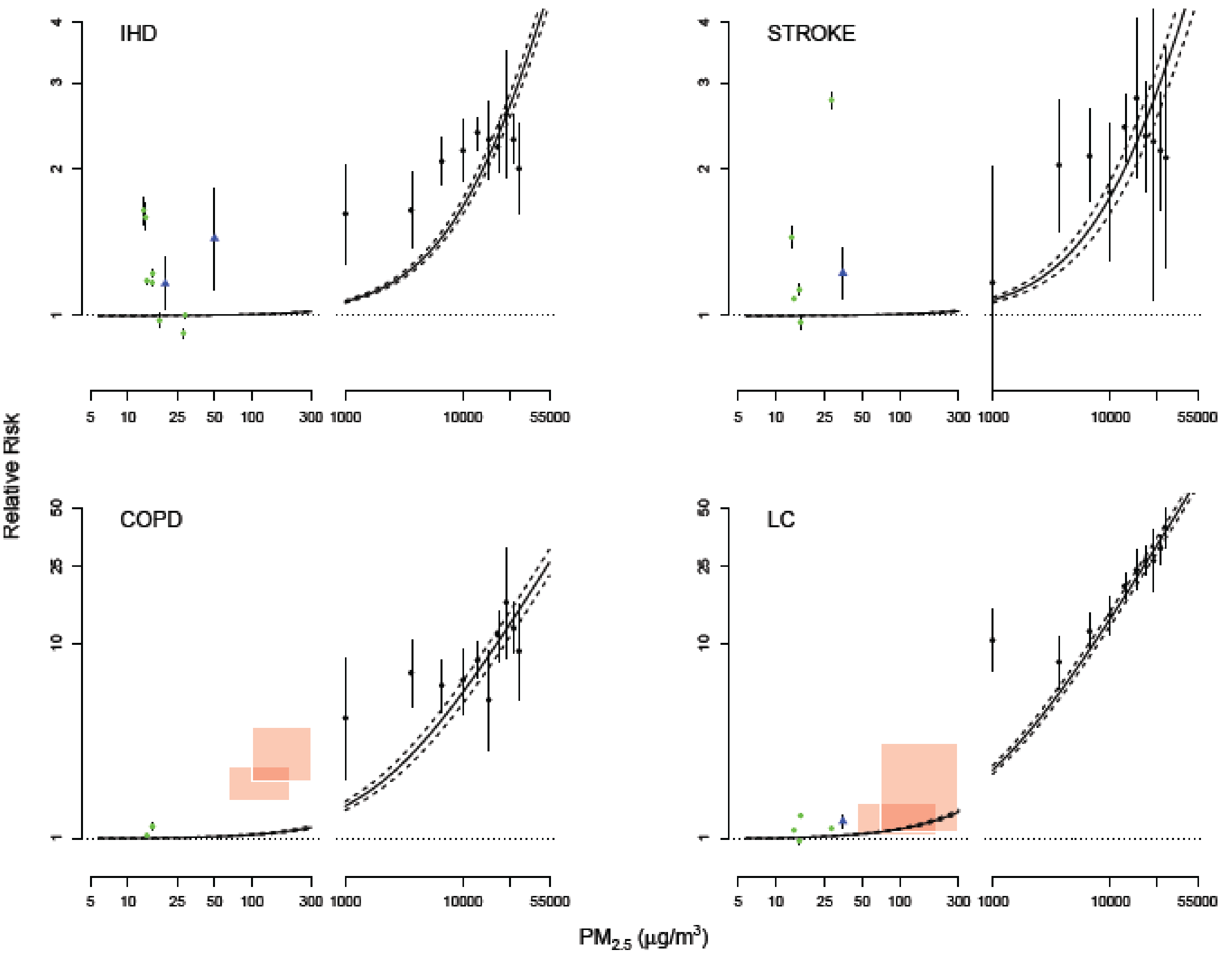

Figure S6. Predicted values of *Linear* model (solid line) and 95% confidence interval (dashed line) for ischemic heart disease (IHD), stroke, chronic obstructive pulmonary disease (COPD), and lung cancer (LC) mortality. Type-specific relative risks (RR) (points) and 95% confidence intervals (error bars) also presented. Green dots represent AAP cohort study RRs, blue triangles represent pooled SHS RRs, and black dots represent AS RRs. Shaded boxes for COPD and LC mortality represent uncertainty (height) and exposure contrast (width) of RR HAP estimates for males and females separately. $PM_{2.5}$ concentrations are on the logarithmic (base 10) scale on x-axis.

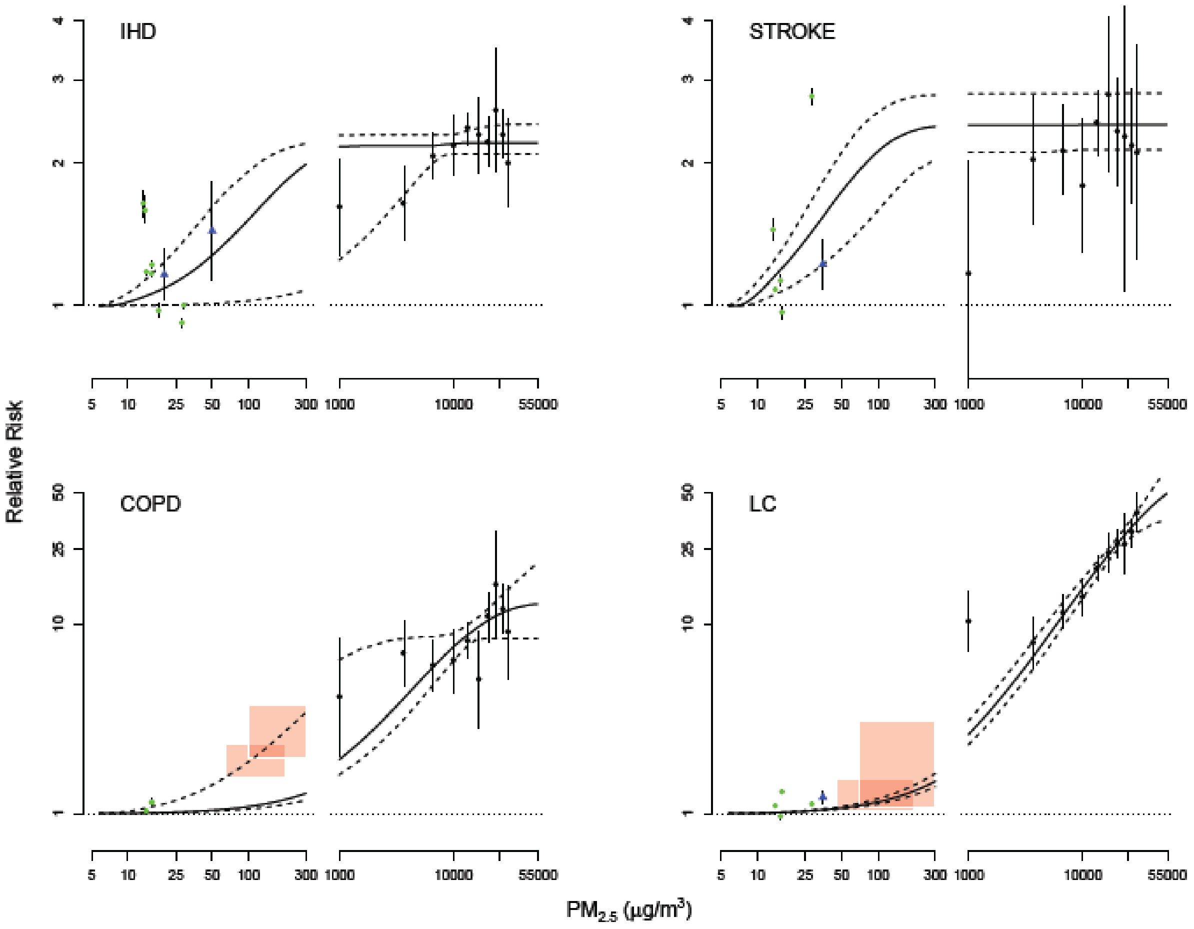

Figure S7. Predicted values of *Exp* model (solid line) and 95% confidence interval (dashed line) for ischemic heart disease (IHD), stroke, chronic obstructive pulmonary disease (COPD), and lung cancer (LC) mortality. Type-specific relative risks (RR) (points) and 95% confidence intervals (error bars) also presented. Green dots represent AAP cohort study RRs, blue triangles represent pooled SHS RRs, and black dots represent AS RRs. Shaded boxes for COPD and LC mortality represent uncertainty (height) and exposure contrast (width) of RR HAP estimates for males and females separately. $PM_{2.5}$ concentrations are on the logarithmic (base 10) scale on x-axis.

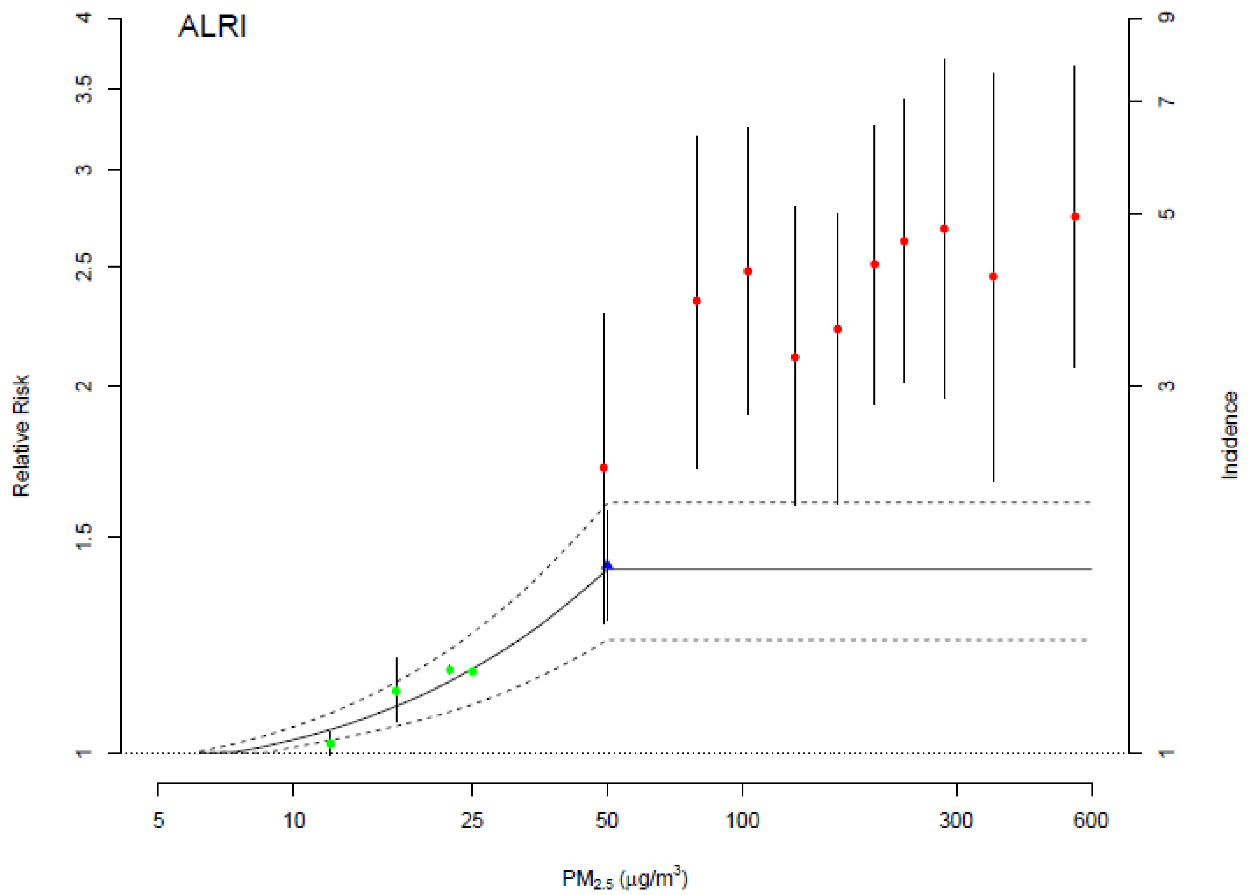

Figure S8. Predicted values of *Lin50* model (solid line) and 95% confidence interval (dashed line) for acute lower respiratory infection infants (ALRI). Type-specific relative risks (RR) (points) and 95% confidence intervals (error bars) also presented. Green dots represent AAP cohort study RRs, blue triangle represents pooled SHS RRs, and red dots represent household air pollution (HAP) RRs. Right hand y-axis represents HAP incidence rates. are $PM_{2.5}$ concentrations are on the logarithmic (base 10) scale on x-axis.

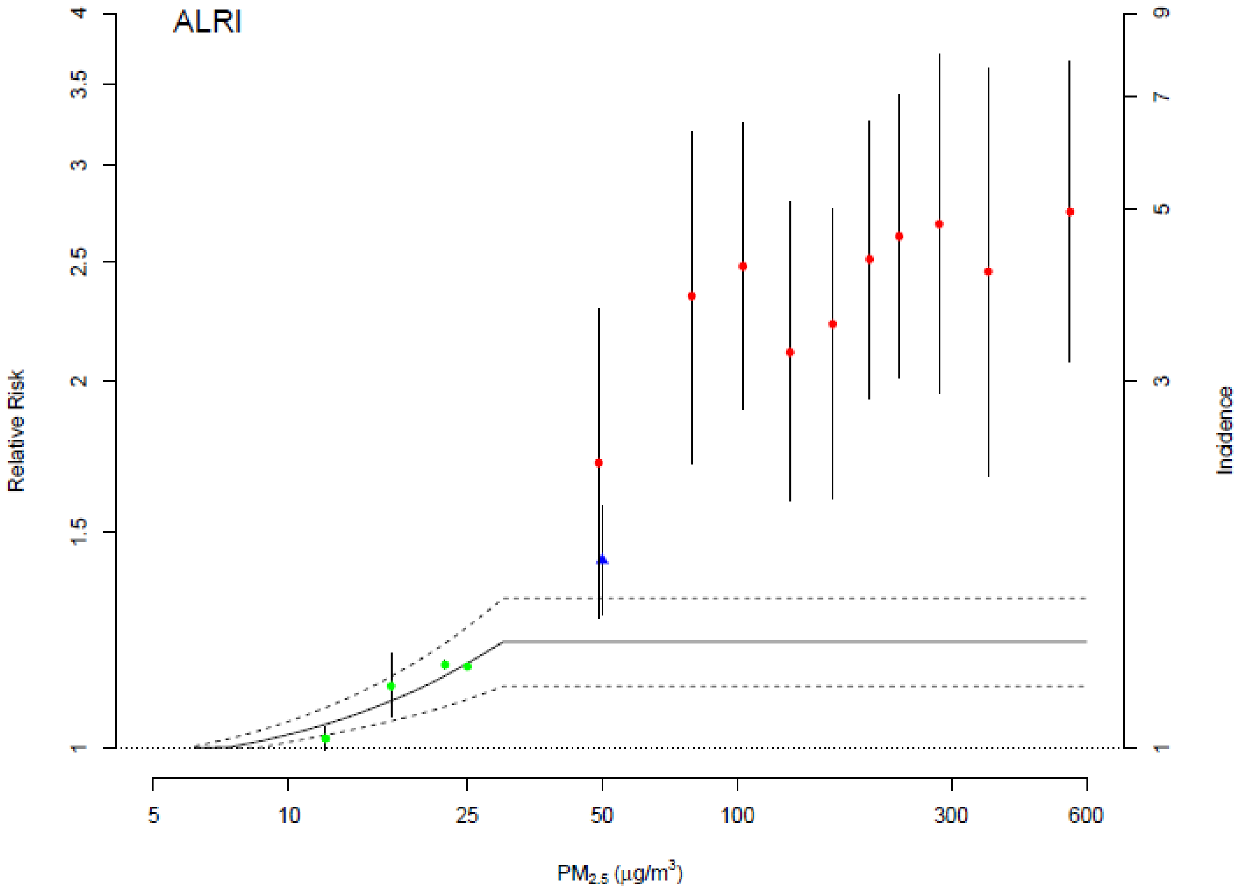

Figure S9. Predicted values of *Lin30* model (solid line) and 95% confidence interval (dashed line) for acute lower respiratory infection infants (ALRI). Type-specific relative risks (RR) (points) and 95% confidence intervals (error bars) also presented. Green dots represent AAP cohort study RRs, blue triangle represents pooled SHS RRs, and red dots represent household air pollution (HAP) RRs. Right hand y-axis represents HAP incidence rates. $PM_{2.5}$ concentrations are on the logarithmic (base 10) scale on x-axis.

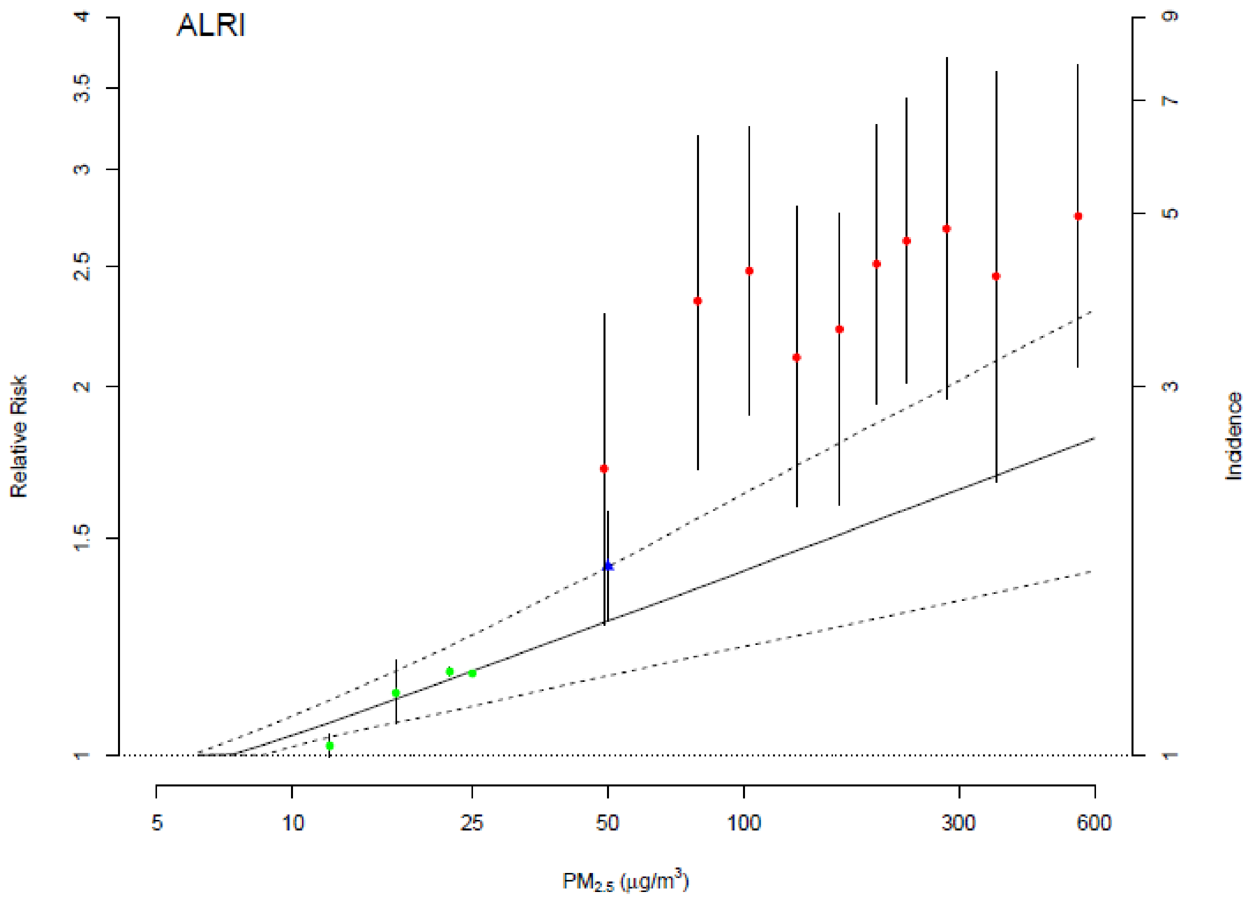

Figure S10. Predicted values of *Log* model (solid line) and 95% confidence interval (dashed line) for acute lower respiratory infection infants (ALRI). Type-specific relative risks (RR) (points) and 95% confidence intervals (error bars) also presented. Green dots represent AAP cohort study RRs, blue triangle represents pooled SHS RRs, and red dots represent household air pollution (HAP) RRs. Right hand y-axis represents HAP incidence rates. $PM_{2.5}$ concentrations are on the logarithmic (base 10) scale on x-axis.

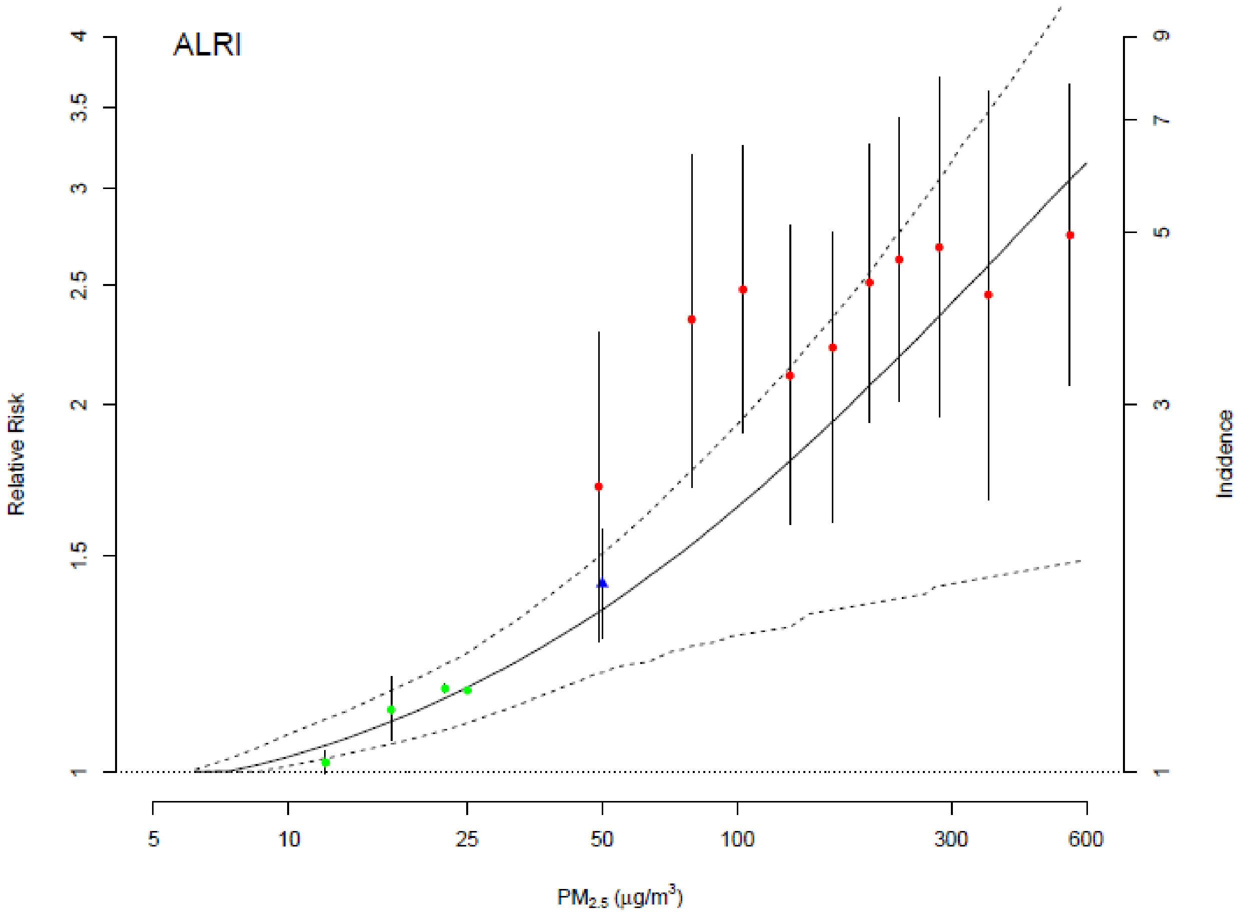

Figure S11. Predicted values of *Log2* model (solid line) and 95% confidence interval (dashed line) for acute lower respiratory infection infants (ALRI). Type-specific relative risks (RR) (points) and 95% confidence intervals (error bars) also presented. Green dots represent AAP cohort study RRs, blue triangle represents pooled SHS RRs, and red dots represent household air pollution (HAP) RRs. Right hand y-axis represents HAP incidence rates. $PM_{2.5}$ concentrations are on the logarithmic (base 10) scale on x-axis.

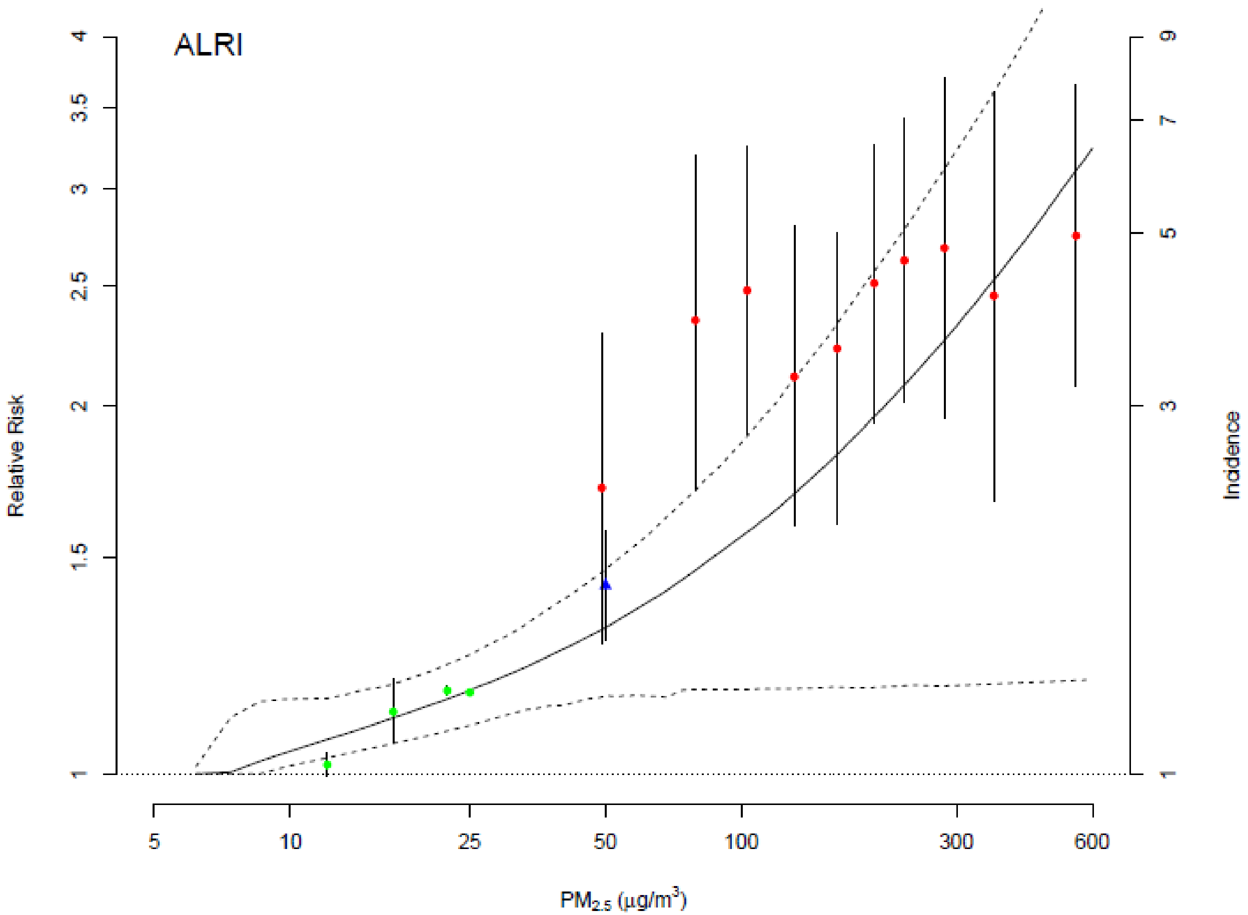

Figure S12. Predicted values of *Power* model (solid line) and 95% confidence interval (dashed line) for acute lower respiratory infection infants (ALRI). Type-specific relative risks (RR) (points) and 95% confidence intervals (error bars) also presented. Green dots represent AAP cohort study RRs, blue triangle represents pooled SHS RRs, and red dots represent household air pollution (HAP) RRs. Right hand y-axis represents HAP incidence rates. $PM_{2.5}$ concentrations are on the logarithmic (base 10) scale on x-axis.

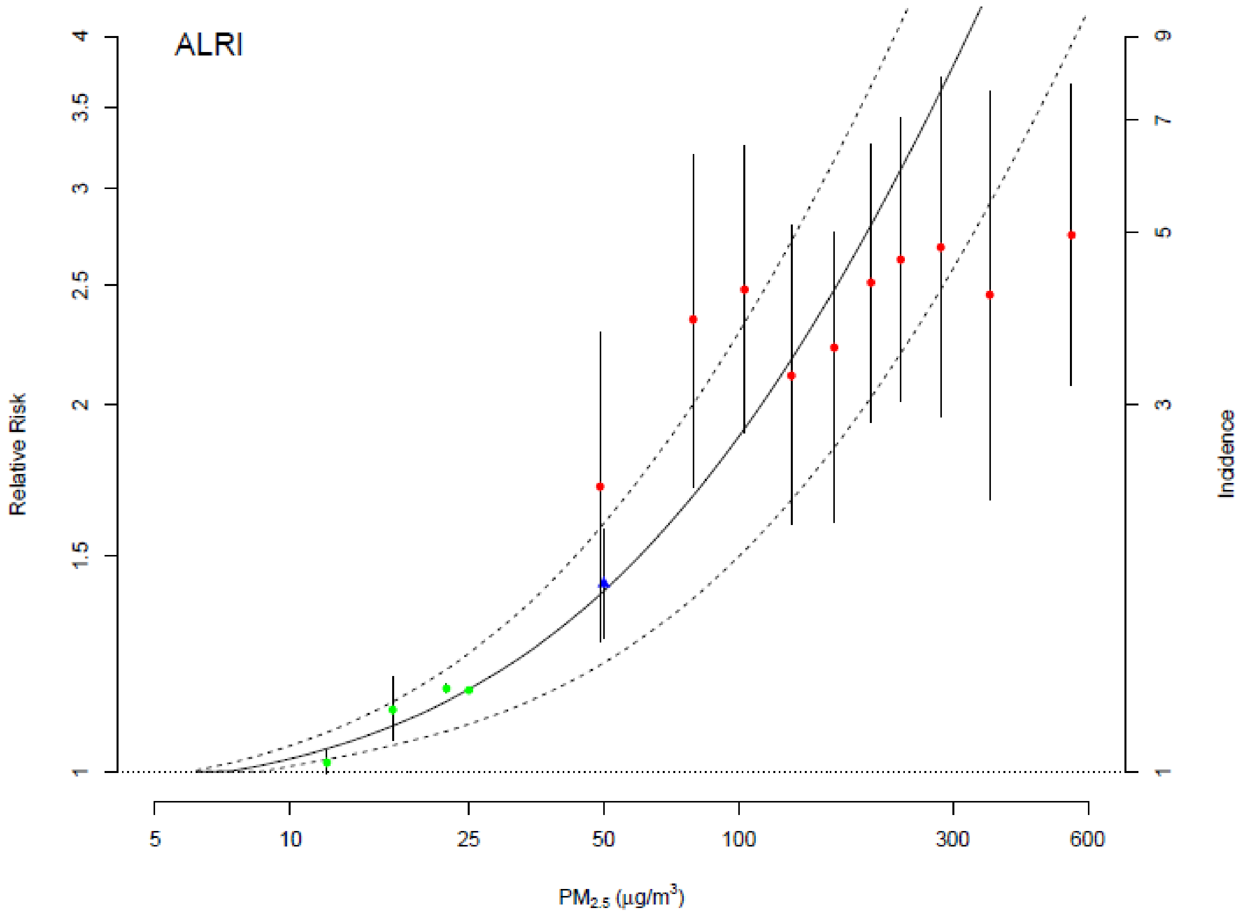

Figure S13. Predicted values of *Linear* model (solid line) and 95% confidence interval (dashed line) for acute lower respiratory infection infants (ALRI). Type-specific relative risks (RR) (points) and 95% confidence intervals (error bars) also presented. Green dots represent AAP cohort study RRs, blue triangle represents pooled SHS RRs, and red dots represent household air pollution (HAP) RRs. Right hand y-axis represents HAP incidence rates. $PM_{2.5}$ concentrations are on the logarithmic (base 10) scale on x-axis.

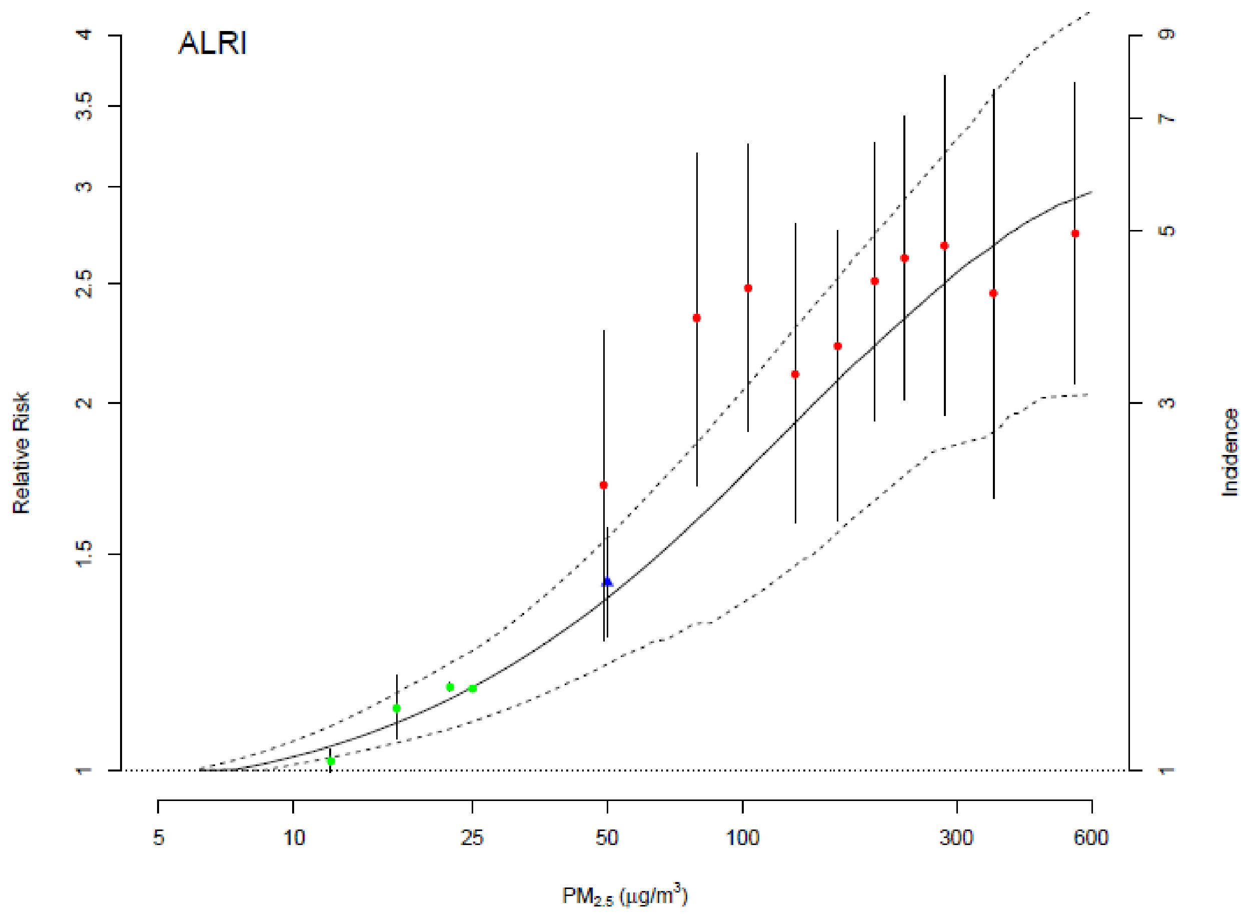

Figure S14. Predicted values of *Exp* model (solid line) and 95% confidence interval (dashed line) for acute lower respiratory infection infants (ALRI). Type-specific relative risks (RR) (points) and 95% confidence intervals (error bars) also presented. Green dots represent AAP cohort study RRs, blue triangle represents pooled SHS RRs, and red dots represent household air pollution (HAP) RRs. Right hand y-axis represents HAP incidence rates. $PM_{2.5}$ concentrations are on the logarithmic (base 10) scale on x-axis.

Table S2. AIC and BIC by outcome and model form.

Outcome	Model Form	AIC	BIC
LC	Exp	-10154.8	-10150.6
LC	Power	-10142.6	-10138.4
LC	Log	-10058.6	-10056.5
LC	Lin	-10151.6	-10149.5
LC	Lin50	-9926.08	-9924
LC	IER	-10250	-10243.8
LC	Lin30	-9978.5	-9976.43
LC	Log2	-10173.8	-10169.6
IHD	Exp	-4695.4	-4692.35
IHD	Power	-4719.88	-4716.83
IHD	Log	-4700.36	-4698.84
IHD	Lin	-4678.08	-4676.56
IHD	Lin50	-4698.39	-4696.86
IHD	IER	-4752.9	-4748.32
IHD	Lin30	-4698.01	-4696.49
IHD	Log2	-4697.99	-4694.94
STROKE	Exp	-4153.66	-4150.55
STROKE	Power	-4133.08	-4129.97
STROKE	Log	-4148.31	-4146.76
STROKE	Lin	-4124.57	-4123.01
STROKE	Lin50	-4128.61	-4127.05
STROKE	IER	-4221.2	-4216.53
STROKE	Lin30	-4123.09	-4121.54
STROKE	Log2	-4140.35	-4137.24
COPD	Exp	-741.901	-740.485
COPD	Power	-757.671	-756.255
COPD	Log	-707.757	-707.049

Outcome	Model Form	AIC	BIC
COPD	Lin	-741.821	-741.113
COPD	Lin50	-692.504	-691.796
COPD	IER	-747.208	-745.084
COPD	Lin30	-704.453	-703.744
COPD	Log2	-749.581	-748.165
ALRI	Exp	-13894.9	-13890.3
ALRI	Power	-13906	-13901.5
ALRI	Log	-13893.4	-13891.1
ALRI	Lin	-13889.2	-13886.9
ALRI	Lin50	-13894.1	-13891.8
ALRI	IER	-13951	-13944.2
ALRI	Lin30	-13893	-13890.7
ALRI	Log2	-13915	-13910.4

Characterizing Uncertainty

We used a simulation approach to estimate uncertainty for both the risk function and PAF based on uncertainty in several components of the relative risk function and PAF.

Uncertainty in the estimates of (α, β, δ)

We first determine the logarithm of the relative risk estimates $\{\hat{r}_1^{(s)},\ldots,\hat{r}_{K_s}^{(s)}, s = 1,\ldots,S\}$ denoted by $\{\hat{\beta}_1^{(s)},\ldots,\hat{\beta}_{K_s}^{(s)}, s = 1,\ldots,S\}$. Further denote the standard error of $\{\hat{\beta}_1^{(s)},\ldots,\hat{\beta}_{K_s}^{(s)}, s = 1,\ldots,S\}$ by $\{\hat{v}_1^{(s)},\ldots,\hat{v}_{K_s}^{(s)}, s = 1,\ldots S\}$. We then generate 1000 realizations of the log-relative risks assuming a normal distribution with mean $\{\hat{\beta}_1^{(s)},\ldots,\hat{\beta}_{K_s}^{(s)}, s = 1,\ldots,S\}$ and standard deviation $\{\hat{v}_1^{(s)},\ldots,\hat{v}_{K_s}^{(s)}, s = 1,\ldots,S\}$ and take their exponents. One thousand estimates of (α, β, δ) are obtained for each set of simulated RRs. We have found in practice that in a small percentage of cases (< 5%) the estimation routine does not converge due to a set of simulated RR that are not consistent with the risk model form. We continue to simulate risks until 1000 estimates of (α, β, δ) are obtained.

Uncertainty in the exposure estimate of $PM_{2.5}$

Estimates of $PM_{2.5}$ and their associated uncertainty are described in detail by Brauer et al. (2011). In summary, the average of a satellite-based remote sensing estimate and an atmospheric model were constructed for each 0.1^o by 0.1^o grid. This summary estimate was calibrated to available ground based monitoring data. The estimate of uncertainty was obtained based on the residual error estimate of the calibration regression equation. Uncertainty in both the remote sensing estimates and the atmospheric model are intrinsically incorporated into our uncertainty estimate based on the residual error of these two $PM_{2.5}$ prediction approaches to the ground data.

Uncertainty in the counterfactual concentration

We define the counterfactual concentration as the lowest exposure level at which we have a reasonable degree of confidence in the range of concentrations to which our outdoor air pollution RR estimates apply. Specifically, we define the counterfactual concentration z_{cf} as a uniform random variable with lower bound defined by the minimum concentration observed in the American Cancer Society Cancer Prevention II cohort (Krewski et al. 2009) of 5.8μg/m^3 and an upper bound defined by the 5th percentile of 8.8 μg/m^3. One thousand draws from this uniform distribution were taken and sequentially applied to the 1000 draws of the source specific RRs in order to estimate (α, β, δ) 1000 times.

Uncertainty in the population attributable risk

For each grid cell within each country a concentration value was independently drawn from the $PM_{2.5}$ uncertainty distribution. Based on the randomly selected concentration in a grid cell, 1000 estimates of (α, β, δ) and z_{cf} were determined and from these values 1000 values 1000 RR_{IER} values were estimated. Then 1000 population-weighted RRs were calculated for each country using the grid cell specific RR_{IER} values. Finally 1000 country-specific PAFs were determined. The mean of these 1000 PAF values was used as our central estimate and the 2.5% and 97.5% values among the 1000 PAFs determined were used to form the lower and upper confidence intervals respectively.

Age-modification risk models for ischemic heart disease and stroke mortality

Epidemiologic studies of risk factors for both IHD and stroke indicate that the RR declines with the logarithm of age, reaching 1 between 100 and 120 (Singh et al. 2013). We thus modified the

type-specific RR for both IHD and stroke mortality using a linear regression model of the logarithm of the median age at death for each study with intercept equal to 1 at age 110. The slope of the regression line was estimated from a meta-analysis of several risk factors (Singh et al. 2013). We applied this age-modification to the RRs and fit the IER model for each age group separately. We compared the country specific estimated PAFs using the age-modified models to those models using age independent data.

Age-modified RR_{IER} curves are displayed for IHD and stroke mortality in Figure S15 (top panels) with generally decreasing risk with increasing age. The country-specific PAFs based on risk models not modified by age and those in which age-modification models were used for both IHD and stroke mortality are presented in Figure S15 (bottom panels). Incorporation of age-modification risk models tends to slightly decrease the PAF estimates.

We compared the distributions of country-specific estimated PAFs for the eight RR models for the five health outcomes (Figure S15). The PAFs were clearly sensitive to the risk model with large variations observed between model specifications for the four causes of death but similar predictions for ALRI. The linear risk model yielded the lowest PAFs for all five health outcomes but the relative ranking in PAFs among models was dependent the cause of death.

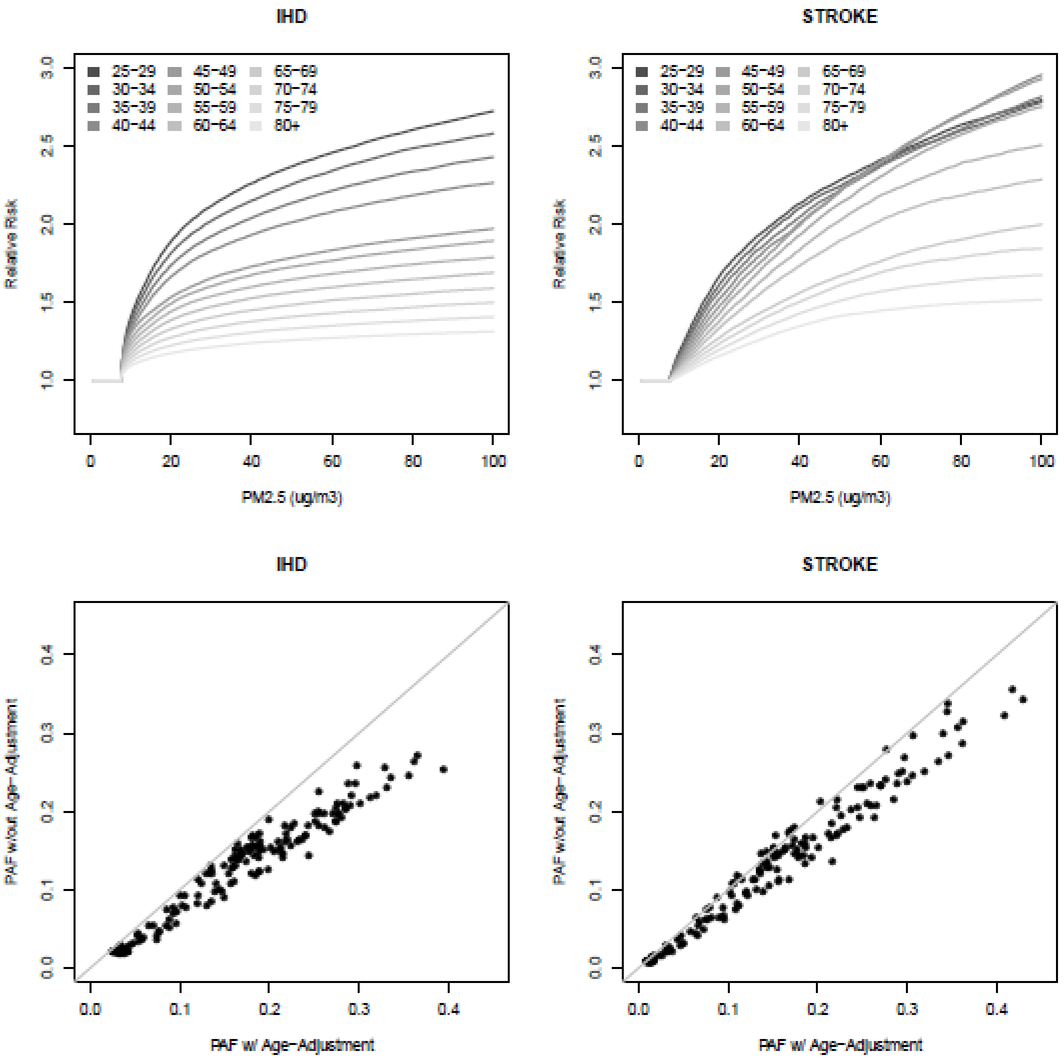

Figure S15. Integrated exposure-response (IER) curves for ischemic heart disease (IHD) (upper left hand panel) and stroke (upper right hand panel) by age group. Comparision of country-specific population attributable risk (PAF) with (y-axis) and without (x-axis) age adjustment for IHD (bottom left panel) and stroke (bottom right panel) with 1:1 line.

References

Akiba S, Kato H, Blot WJ. Passive smoking and lung cancer among Japanese women. Cancer Research 1986;46(9):4804–7.

Baker RJ, Hertz-Picciotto I, Dostal M, Keller JA, Nozicka J, Kotesovec F, Dejmek J, Loomis D, Sram RJ: Coal home heating and environmental tobacco smoke in relation to lower respiratory illness in Czech children, from birth to 3 years of age. Environ Health Perspect 2006, 114:1126-1132.

Beelen R, Hoek G, van den Brandt AP, Goldbohm RA, Fischer P, Schouten LJ, Jerrett M, Hughes E, Armstrong B, Brunekreef B. 2008. Long-Term Effects of Traffic-Related Air Pollution on Mortality in a Dutch Cohort (NLCS-AIR Study). Environ. Health Perspect. 116:196–202.

Blizzard L, Ponsonby A-L, Dwyer T, Venn A, Cochrane JA: Parental smoking and infant respiratory infection: how important is not smoking in the same room with the baby? Am J Public Health 2003, 93:482-488

Boffetta P, Ahrens W, Nyberg F, Mukeria A, Brüske- Hohlfeld I, Fortes C, Constantinescu V, Simonato L, Batura-Gabryel H, Lea S, et al. Exposure to environmental tobacco smoke and risk of adenocarcinoma of the lung. International Journal of Cancer 1999;83(5):635–9.

Bonu S, Rani M, Jha P, Peters DH, Nguyen SN: Household tobacco and alcohol use, and child health: an exploratory study from India. Health Policy 2004, 70:67-83.

Brauer M, Hoek G, Van Vliet P, Meliefste K, Fischer PH, Wijga A, et al. 2002. Air pollution from traffic and the development of respiratory infections and asthmatic and allergic symptoms in children. Am J Respir Crit Care Med 166:1092–1098.

Brauer M, Amann M, Burnett RT, Cohen A, Denterner F, Ezzati M, Henderson SB, Krzyzanowski M, Martin RV, Van Dingenen R, van Donkelaar A, Thurston GD. 2012. Exposure assessment for estimation of the global burden of disease attributable to outdoor air pollution. Environ. Sci. & Technol. 46:652−660.

Broor S, Pandey RM, Ghosh M, Maitreyi RS, Lodha R, Singhal T, Kabra SK: Risk factors for severe acute lower respiratory tract infection in under-five children. Indian Pediatr 2001, 38:1361-1369.

Brownson RC, Alavanja MCR, Hock ET, Loy TS. Passive smoking and lung cancer in nonsmoking women. American Journal of Public Health 1992; 82(11):1525–30.

Brownson RC, Reif JS, Keefe TJ, Ferguson SW, Pritzl JA. Risk factors for adenocarcinoma of the lung. American Journal of Epidemiology 1987;125(1):25–34.

Buffler PA, Pickle LW, Mason TJ, Contant C. The causes of lung cancer in Texas. In: Mizell M, Correa P, editors. Lung Cancer: Causes and Prevention. Deerfield Beach (FL): VerlagChemie International, 1984:83–99.

Butler TL. The relationship of passive smoking to various health outcomes among Seventh-day Adventists in California [dissertation]. Los Angeles: University of California, 1988.

Cardenas VM, Thun MJ, Austin H, Lally CA, Clark WS, Greenberg RS, Heath CW Jr. Environmental tobacco smoke and lung cancer mortality in the American Cancer Society's Cancer Prevention Study II. Cancer Causes and Control 1997;8(1):57–64.

Chan WC, Fung SC. Lung cancer in non-smokers in Hong Kong. In: Grundmann E, Clemmesen J, Muir CS, editors. Geographical Pathology in Cancer Epidemiology. Cancer Campaign. Vol. 6. New York: Gustav Fischer Verlag, 1982:199–202.

Chen LH, Knutsen SF, Shavlik D, Beeson LW, Petersen F, Ghamsary M, Abbey D. 2005. The Association between Fatal Coronary Heart Disease and Ambient Particulate Air Pollution: Are Females at Greater Risk? Environ. Health Perspect. 113:1723–1729.

Chen Y: Environmental tobacco smoke, low birth weight, and hospitalization for respiratory disease. Am J Respir Crit Care Med 1994, 150:54-58.

Ciruzzi M, Pramparo P, Esteban O, Rozlosnik J, Tartaglione J, Abecasis B, César J, De Rosa J, Paterno C, Schargrodsky H. Case-control study of passive smoking at home and risk of acute myocardial infarction. *Journal of the American College of Cardiology* 1998;31(4):797–803.

Cohen A, Anderson HR, Ostro B, Pandey K, Krzyzanowski M, Kunzli N et al. 2004. Urban air pollution. In: Ezzati M, Lopez A, Rodgers A, Murray CJL, eds. *Comparative quantification of health risks: global and regional burden of disease attributable to selected major risk factors.* Geneva, World Health Organization:1353–1433.

Correa P, Pickle LW, Fontham E, Lin Y, Haenszel W. Passive smoking and lung cancer. Lancet 1983;2(8350):595–7.

Duijts L, Jaddoe VWV, Hofman A, Steegers EAP, Mackenbach JP, de Jongste JC, Moll HA: Maternal smoking in pre-natal and early post-natal life and the risk of respiratory tract infections in infancy. The Generation R study. Eur J Epidemiol 2008, 23:547-555.

Ekwo EE, Weinberger MM, Lachenbruch PA, Huntley WH: Relationship of parental smoking and gas cooking to respiratory disease in children. Chest 1983, 84:662-668.

Etiler N, Velipasaoglu S, Aktekin M: Incidence of acute respiratory infections and the relationship with some factors in infancy in Antalya, Turkey. PediatrInt 2002, 44:64-69.

Ferris BG Jr, Ware JH, Berkey CS, Dockery DW, Spiro A, Speizer FE: Effects of passive smoking on health of children. Environ Health Perspect 1985, 62:289-295.

Fontham ETH, Correa P, Reynolds P, Wu-Williams A, Buffler PA, Greenberg RS, Chen VW, Alterman T, Boyd P, Austin DF, et al. Environmental tobacco smoke and lung cancer in nonsmoking women: a multicenter study. Journal of the American Medical Association 1994;271(22):1752–9.

Forastiere F, Corbo GM, Michelozzi P, Pistelli R, Agabiti N, Brancato G, Ciappi G, Perucci CA: Effects of environment and passive smoking on the respiratory health of children. Int J Epidemiol 1992, 21:66-73.

Gardner G, Frank AL, Taber LH: Effects of social and family factors on viral respiratory infection and illness in the first year of life. J Epidemiol Community Health 1984, 38:42-48.

Garfinkel L, Auerbach O, Joubert L. Involuntary smoking and lung cancer: a case-control study. Journal of the National Cancer Institute 1985;75(3):463–9.

Gao Y-T, Blot WJ, Zheng W, Ershow AG, Hsu CW, Levin LI, Zhang R, Fraumeni JF Jr. Lung cancer among Chinese women. International Journal of Cancer 1987;40(5):604–9.

Geng G-Y, Liang ZH, Zhang A-Y, Wu GL. On the relationship between smoking and female lung cancer. In: Aoki M, Hisamichi S, Tominaga S, editors. Smoking and Health 1987. Proceedings of the 6th World Conference on Smoking and Health, Nov 9–12, 1987; Tokyo. Amsterdam: Elsevier Science, 1988:483–6.

Gillis C, Hole D, Hawthorne V et al. The effect of environmental tobacco smoke in two urban communities in the west of Scotland Eur J Respir Dis Suppl 1984;133:121–6.

Glymour M, Defries T, Kawachi I et al. Spousal smoking and incidence of first stroke: the Health and Retirement Study. Am J Prev Med 2008;35(3):245–8.

Hassan MK, Al-Sadoon I: Risk factors for severe pneumonia in children in Basrah. Trop Doct 2001, 31:139-141.

He Y, Lam TH, Li LS, Du RY, Jia GL, Huang JY, Zheng JS. Passive smoking at work as a risk factor for coronary heart disease in Chinese women who have never smoked. *British Medical Journal* 1994;308(6925):380–4.

Hertz-Picciotto I, Baker RJ, Yaw PS, Dostal M, Joad JP, Lipsett M, et al. 2007. Early childhood lower respiratory illness and air pollution. Environ Health Perspect 115:1510–1526. doi:10.1289/ehp.9617

Hill SE, Blakely T, Kawachi I et al. Mortality among lifelong nonsmokers exposed to secondhand smoke at home: Cohort data and sensitivity analyses. Am J Epidemiol 2007;165:530–40.

Hirayama T. Passive smoking [letter]. *New Zealand Medical Journal* 1990;103(883):54.

Hirayama T. Non-smoking wives of heavy smokers have a higher risk of lung cancer: a study from Japan. British Medical Journal 1981;282(6259):183–5.

Hole DJ, Gillis CR, Chopra C, Hawthorne VM. Passive smoking and cardiorespiratory health in a general population in the west of Scotland. *British Medical Journal* 1989;299(6696):423–7.

Humble CG, Samet JM, Pathak DR. Marriage to a smoker and lung cancer risk. American Journal of Public Health 1987;77(5):598–602.

Iribarren C, Darbinian J, Klatsky AL et al. Cohort study of exposure to environmental tobacco smoke and risk of first ischemic stroke and transient ischemic attack. Neuroepidemiology 2004;23(1–2):38–44.

Inoue R, Hirayama T. Passive smoking and lung cancer in women. In: Aoki M, Hisamichi S, Tominaga S, editors. Smoking and Health 1987. Proceedings of the 6th World Conference on Smoking and Health, Nov 9–12, 1987; Tokyo. Amsterdam: Elsevier Science, 1988:283–5.

Jefferis BJ, Lawlor DA, Ebrahim S et al. Cotinine assessed secondhand smoke exposure and risk of cardiovascular disease in older adults. Heart 2010;96(11):854–9.

Johnson KC, Hu J, Mao Y, the Canadian Cancer Registries Epidemiology Research Group. Lifetime residential and workplace exposure to environmental tobacco smoke and lung cancer in never-smoking women, Canada 1994–97. International Journal of Cancer 2001;93(6):902–6.

Kabat GC, Stellman SD, Wynder EL. Relation between exposure to environmental tobacco smoke and lung cancer in lifetime nonsmokers. American Journal of Epidemiology 1995;142(2):141–8.

Kabat GC, Wynder EL. Lung cancer in nonsmokers. Cancer 1984;53(5):1214–21.

Kalandidi A, Katsouyanni K, Voropoulou N, Bastas G, Saracci R, Trichopoulos D. Passive smoking and diet in the etiology of lung cancer among nonsmokers. Cancer Causes and Control 1990;1(1):15–21.

Karr C, Lumley T, Schreuder A, Davis R, Larson T, Ritz B, et al. 2007. Effects of subchronic and chronic exposure to ambient air pollutants on infant bronchiolitis. Am J Epidemiol 165 (5):553–560. doi:10.1093/aje/kwk032.

Karr CJ, Rudra CB, Miller KA, Gould TR, Larson T, Sathyanarayana S, 2009. Infant exposure to fine particulate matter and traffic and risk of hospitalization for RSV bronchiolitis in a region with lower ambient air pollution. Environ Res 109 (3):321–327. doi:10.1016/j.envres.2008.11.006.

Ko Y-C, Lee CH, Chen MJ, Huang CC, Chang WY, Lin HJ, Wang HZ, Chang PY. Risk factors for primary lung cancer among non-smoking women in Taiwan. International Journal of Epidemiology 1997; 26(1):24–31.

Koch A, Molbak K, Homoe P, Sorensen P, Hjuler T, Olesen ME, Pejl J, Pedersen FK, Olsen OR, Melbye M: Risk factors for acute respiratory tract infections in young Greenlandic children. Am J Epidemiol 2003, 158:374-384.

Koo LC, Ho JH-C, Saw D, Ho C-Y. Measurements of passive smoking and estimates of lung cancer risk among non-smoking Chinese females. International Journal of Cancer 1987;39(2):162–9.

Krewski D, Jerrett M, Burnett RT, Ma R, Hughes E, Shi Y, et al. 2009. Extended Follow-Up and Spatial Analysis of the American Cancer Society Study Linking Particulate Air Pollution and Mortality. HEI Research Report 140. Boston, MA: Health Effects Institute. Available: http://pubs.healtheffects.org/getfile.php?u=478.

Kristensen IA, Olsen J: Determinants of acute respiratory infections in Soweto--a population-based birth cohort. S Afr Med J 2006, 96:633-640.

La Vecchia C, D'Avanzo B, Franzosi MG, Tognoni G. Passive smoking and the risk of acute myocardial infarction. *Lancet* 1993;341(8843):505–6.

Lam TH, Kung ITM, Wong CM, Lam WK, Kleevens JWL, Saw D, Hsu C, Seneviratne S, Lam SY, Lo KK, et al. Smoking, passive smoking and histological types in lung cancer in Hong Kong Chinese women. British Journal of Cancer 1987;56(5):673–8.

Lee C-H, Ko Y-C, Goggins W, Huang J-J, Huang M-S, Kao E-L, Wang H-Z. Lifetime environmental exposure to tobacco smoke and primary lung cancer of non-smoking Taiwanese women. International Journal of Epidemiology 2000;29(2):224–31.

Lepeule J, Ladon F, Dockery D, Schwartz J. (2012). Chronic exposure to fine particles and mortality: an extended follow-up of the Harvard Six Cities Study from 1974 to 2009. Environ. Health Perspect. 120:965-970.

Lim SS, Vos T, Flaxman AD, Danaei G, Shibaya K, Adair-Rahani H, et al. (2012). A comparative risk assessment of burden of disease and injury attributable to 67 risk factors and risk factor clusters in 21 regions, 1990–2010: a systematic analysis for the Global Burden of Disease Study 2010. Lancet 380:2224-2260.

Lipsett MJ, Ostro BD, Reynolds P, Goldberg D, Hertz A, Jerrett M, et al. 2011. Long-term Exposure to Air Pollution and Cardiorespiratory Disease in the California Teachers Study Cohort. Am J Respir Crit Care Med. 184: 828-83.

Liu Q, Sasco AJ, Riboli E, Hu MX. Indoor air pollution and lung cancer in Guangzhou, People's Republic of China. American Journal of Epidemiology 1993;137(2):145–54.

Liu Z, He X, Chapman RS. Smoking and other risk factors for lung cancer in Xuanwei, China. International Journal of Epidemiology 1991;20(1):26–31.

Margolis PA, Keyes LL, Greenberg RA, Bauman KE, LaVange LM: Urinary cotinine and parent history (questionnaire) as indicators of passive smoking and predictors of lower respiratory illness in infants. PediatrPulmonol 1997, 23:417-423.

Miller KA, Siscovick DS, Sheppard L, Shepherd K, Sullivan JH, Anderson GL, et al. 2007. Long-Term Exposure to Air Pollution and Incidence of Cardiovascular Events in Women. N. Engl. J. Med. 356:447-58.

Nuesslein TG, Beckers D, Rieger CH: Cotinine in meconium indicates risk for early respiratory tract infections. Hum ExpToxicol 1999, 18:283-290.

Nyberg F, Isaksson I, Harris JR, Pershagen G. Misclassification of smoking status and lung cancer risk from environmental tobacco smoke in neversmokers. Epidemiology 1997;8(3):304–9.

Ogston SA, Florey CD, Walker CH: Association of infant alimentary and respiratory illness with parental smoking and other environmental factors. J Epidemiol Community Health 1987, 41:21-25.

Ogston SA, Florey CD, Walker CH: The Tayside infant morbidity and mortality study: effect on health of using gas for cooking. BMJ 1985, 290:957-960.

Ostro B. 2004. Outdoor Air Pollution: Assessing the Environmental Burden of Disease at National and Local Levels. Environmental Burden of Disease Series, No. 5. Pruss-Ustun A, Campbell-Lendrum, D, Corvalan C, Woodward A., eds. World Health Organization: Geneva. Available: http://www.who.int/quantifying_ehimpacts/publications/ebd5.pdf.

Pedreira FA, Guandolo VL, Feroli EJ, Mella GW, Weiss IP: Involuntary smoking and incidence of respiratory illness during the first year of life. Pediatrics 1985, 75:594-597.

Pershagen G, Hrubec Z, Svensson C. Passive smoking and lung cancer in Swedish women. American Journal of Epidemiology 1987;125(1):17–24.

Pope CA III, Burnett RT, Krewski D, Jerrett M, Shi Y, Calle EE, et al. 2009. Cardiovascular Mortality and Exposure to Fine Particulate Matter from Air Pollution and Cigarette Smoke: Shape of the Exposure-Response Relationship. Circulation 120:941-948.

Pope CA III, Burnett RT, Turner MC, Cohen A, Krewski D, Jerrett M, Gapstur S, Thun MJ 2011. Lung Cancer and Cardiovascular Disease Mortality Associated with Particulate Matter Exposure from Ambient Air Pollution and Cigarette Smoke: Shape of the Exposure-Response Relationships. Environ. Health Perspect. 119:1616-1621.

Puett RC, Hart JE, Yanosky JD, Paciorek C, Schwartz J, Suh H, et al. 2009. Chronic Fine and Coarse Particulate Exposure, Mortality and Coronary Heart Disease in the Nurses' Health Study. Environ. Health Perspect. 117:1697-1729.

Puett RC, Hart JE, Suh H, Mittleman M, Laden F. 2011. Particulate matter exposures, Mortality, and cardiovascular disease in the Health Professionals Follow-up Study. Environ. Health Perspect. 119:1130-1135.

Qureshi AI, Suri MF, Kirmani JF et al. Cigarette smoking among spouses: Another risk factor for stroke in women. Stroke 2005;36(9):e74–6.

Rosenlund M, Berglind N, Gustavsson A, Reuterwall C, Hallqvist J, Nyberg F, Pershagen G. Environmental tobacco smoke and myocardial infarction among never-smokers in the Stockholm Heart Epidemiology Program (SHEEP). Epidemiology 2001;12(5):558–64.

Rylander E, Pershagen G, Eriksson M, Bermann G: Parental smoking, urinary cotinine, and wheezing bronchitis in children. Epidemiology 1995, 6:289-293.

Sandler DP, Comstock GW, Helsing KJ et al. Deaths from all causes in non-smokers who lived with smokers. Am J Public Health 1989;79(2):163–7.

Shen X-B, Wang G-X, Zhou B-S. Relation of exposure to environmental tobacco smoke and pulmonary adenocarcinoma in non-smoking women: a case control study in Nanjing. Oncology Reports 1998;5(5):1221–3.

Shimizu H, Morishita M, Mizuno K, Masuda T, Ogura Y, Santo M, Nishimura M, Kunishima K, Karasawa K, Nishiwaki K, et al. A case-control study of lung cancer in nonsmoking women. Tohoku Journal of Experimental Medicine 1988;154(4):389–97.

Singh GM, Danaei G, Farzadfar F, et al. 2013. The age-specific quantitative effects of metabolic risk factors on cardiovascular diseases and diabetes: a pooled analysis. PLoS One 8:e65174.

Smith KR, McCracken JP, Weber MW, Hubbard A, Jenny A, Thompso LM, et al. 2011. Effect of reduction in household air pollution on childhood pneumonia in Guatemala (RESPIRE): a randomised controlled trial. Lancet 378:1717–26.

Smith KR, Bruce N, Balakrishnan K, Adair-Rohani H, Balmes J, Chafe Z, et al. 2014. Millions dead: how do we know and what does it mean? Methods used in the Comparative Risk Assessment of Household Air Pollution, Ann Rev of Public Health, 35:27.1.- 27.23 (in press, 2014).

Sobue T. Association of indoor pollution and passive smoking with lung cancer in Osaka, Japan. International Journal of Epidemiology 1990;19(Suppl 1): S62–S66.

Steenland K, Thun M, Lally C, Heath C Jr. Environmental tobacco smoke and coronary heart disease in the American Cancer Society CPS-II cohort. Circulation 1996;94(4):622–8.

Stockwell HG, Goldman AL, Lyman GH, Noss CI, Armstrong AW, Pinkham PA, Candelora EC, Brusa MR. Environmental tobacco smoke and lung cancer risk in nonsmoking women. Journal of the National Cancer Institute 1992;84(18):1417–22.

Suzuki M, Thiem VD, Yanai H, Matsubayashi T, Yoshida LM, Tho LH, Minh TT, Anh DD, Kilgore PE, Ariyoshi K: Association of environmental tobacco smoking exposure with an increased risk of hospital admissions for pneumonia in children under 5 years of age in Vietnam. Thorax 2009, 64:484-489.

Svendsen KH, Kuller LH, Martin MJ, Ockene JK.Effects of passive smoking in the Multiple Risk Factor Intervention Trial. *American Journal of Epidemiology* 1987;126(5):783–95.

Svensson C, Pershagen G, Klominek J. Smoking and passive smoking in relation to lung cancer in women. ActaOncologica 1989;28(5):623–9.

Taylor B, Wadsworth J: Maternal smoking during pregnancy and lower respiratory tract illness in early life. Arch Dis Child 1987, 62:786-791.

Trichopoulos D, Kalandidi A, Sparros L. Lung cancer and passive smoking: conclusion of Greek study [letter]. Lancet 1983;2(8351):677–8.

Victora CG, Fuchs SC, Flores JA, Fonseca W, Kirkwood B: Risk factors for pneumonia among children in a Brazilian metropolitan area. Pediatrics 1994, 93:977-985.

Wang T-J, Zhou B-S, Shi J-P. Lung cancer in nonsmoking Chinese women: a case-control study. Lung Cancer 1996;14(Suppl 1):S93–S98.

Wen W, Shu XO, Gao Y-T et al. Environmental tobacco smoke and mortality in Chinese women who have never smoked: prospective cohort study. BMJ 2006;333(7564):376–00.

Whincup PH, Gilg JA, Emberson JR et al. Passive smoking and risk of coronary heart disease and stroke: prospective study with cotinine measurement. BMJ 2004;329:200–5.

World Health Organization. 2002. World Health Report 2002 - Reducing Risks, Promoting Healthy Life. WHO: Geneva. Available: http://www.who.int/whr/2002/en/whr02_en.pdf .

Wu AH, Henderson BE, Pike MC, Yu MC. Smoking and other risk factors for lung cancer in women. Journal of the National Cancer Institute 1985;74(4):747–51.

Wu-Williams AH, Samet JH. Environmental tobacco smoke: exposure-response relationships in epidemiolgic studies. Risk Analysis 1990;10(1):39–48.

Yamada S, Koizumi A, Iso H et al. Risk factors for fatal subarachnoid hemorrhage: the Japan Collaborative Cohort Study. Stroke 2003;34(12):2781–7.

Zaridze D, Maximovitch D, Zemlyanaya G, Aitakov ZN, Boffetta P. Exposure to environmental tobacco smoke and risk of lung cancer in non-smoking women from Moscow, Russia. International Journal of Cancer 1998;75(3):335–8.
